# Assessment of Natural Language Processing of Electronic Health Records to Measure Goals-of-Care Discussions as a Clinical Trial Outcome

**DOI:** 10.1001/jamanetworkopen.2023.1204

**Published:** 2023-03-02

**Authors:** Robert Y. Lee, Erin K. Kross, Janaki Torrence, Kevin S. Li, James Sibley, Trevor Cohen, William B. Lober, Ruth A. Engelberg, J. Randall Curtis

**Affiliations:** 1 Cambia Palliative Care Center of Excellence at UW Medicine, University of Washington, Seattle; 2 Division of Pulmonary, Critical Care, and Sleep Medicine, Department of Medicine, University of Washington, Seattle; 3 Division of Biomedical and Health Informatics, Department of Biomedical Informatics and Medical Education, University of Washington, Seattle; 4 Department of Biobehavioral Nursing and Health Informatics, University of Washington, Seattle; 5 Department of Global Health, University of Washington, Seattle; 6 Department of Health Systems and Population Health, University of Washington, Seattle

## Abstract

**Importance:**

Many clinical trial outcomes are documented in free-text electronic health records (EHRs), making manual data collection costly and infeasible at scale. Natural language processing (NLP) is a promising approach for measuring such outcomes efficiently, but ignoring NLP-related misclassification may lead to underpowered studies.

**Objective:**

To evaluate the performance, feasibility, and power implications of using NLP to measure the primary outcome of EHR-documented goals-of-care discussions in a pragmatic randomized clinical trial of a communication intervention.

**Design, Setting, and Participants:**

This diagnostic study compared the performance, feasibility, and power implications of measuring EHR-documented goals-of-care discussions using 3 approaches: (1) deep-learning NLP, (2) NLP-screened human abstraction (manual verification of NLP-positive records), and (3) conventional manual abstraction. The study included hospitalized patients aged 55 years or older with serious illness enrolled between April 23, 2020, and March 26, 2021, in a pragmatic randomized clinical trial of a communication intervention in a multihospital US academic health system.

**Main Outcomes and Measures:**

Main outcomes were natural language processing performance characteristics, human abstractor-hours, and misclassification-adjusted statistical power of methods of measuring clinician-documented goals-of-care discussions. Performance of NLP was evaluated with receiver operating characteristic (ROC) curves and precision-recall (PR) analyses and examined the effects of misclassification on power using mathematical substitution and Monte Carlo simulation.

**Results:**

A total of 2512 trial participants (mean [SD] age, 71.7 [10.8] years; 1456 [58%] female) amassed 44 324 clinical notes during 30-day follow-up. In a validation sample of 159 participants, deep-learning NLP trained on a separate training data set identified patients with documented goals-of-care discussions with moderate accuracy (maximal F_1_ score, 0.82; area under the ROC curve, 0.924; area under the PR curve, 0.879). Manual abstraction of the outcome from the trial data set would require an estimated 2000 abstractor-hours and would power the trial to detect a risk difference of 5.4% (assuming 33.5% control-arm prevalence, 80% power, and 2-sided α = .05). Measuring the outcome by NLP alone would power the trial to detect a risk difference of 7.6%. Measuring the outcome by NLP-screened human abstraction would require 34.3 abstractor-hours to achieve estimated sensitivity of 92.6% and would power the trial to detect a risk difference of 5.7%. Monte Carlo simulations corroborated misclassification-adjusted power calculations.

**Conclusions and Relevance:**

In this diagnostic study, deep-learning NLP and NLP-screened human abstraction had favorable characteristics for measuring an EHR outcome at scale. Adjusted power calculations accurately quantified power loss from NLP-related misclassification, suggesting that incorporation of this approach into the design of studies using NLP would be beneficial.

## Key Points

**Question:**

Can natural language processing (NLP) be used to measure clinical trial outcomes?

**Findings:**

In this diagnostic study evaluating the performance, feasibility, and power implications of using deep-learning NLP to measure the outcome of documented goals-of-care discussions in a 2512-patient pragmatic trial, NLP-screened human abstraction measured the outcome with 92.6% sensitivity, substantial savings in abstractor-hours, and minimal loss of power, compared with manual abstraction.

**Meaning:**

The findings suggest that NLP may facilitate measurement of previously inaccessible outcomes in clinical trials and that incorporation of misclassification-adjusted power calculations into the design of studies using NLP may be beneficial.

## Teaser

This diagnostic study evaluates the performance, feasibility, and power implications of using natural language processing to measure outcomes in a randomized clinical trial of a communication intervention among adults with serious illness.

## Introduction

Natural language processing (NLP) of free-text electronic health records (EHRs) presents rich opportunities for measuring outcomes that would otherwise require costly, laborious medical record abstraction.^1,2,3^ However, NLP can introduce inaccuracies and misclassify outcomes, particularly when measuring complex constructs.^4,5,6,7^ Many statistical procedures common in clinical research ignore misclassification, and applying these procedures to imperfectly measured outcomes can lead to underpowered studies and improper estimates.^8,9,10^ In using NLP for clinical research, it is important to implement robust approaches to address NLP-related misclassification in study design and analysis.^9,10^

Researchers in the fields of palliative care and serious illness communication have shown interest in using NLP^7,11,12,13,14^ to measure the occurrence and timing of EHR-documented goals-of-care discussions, an outcome that reflects clinicians’ assessment and documentation of patients’ values, goals, and treatment preferences.^15,16^ This outcome represents a guideline-recommended best practice,^16,17,18,19,20^ an area of ongoing deficiencies,^21,22,23^ and a mediator of delivery of patient-centered care.^16,24,25,26^ However, goals-of-care discussions are difficult to measure from structured EHR data or claims data, and their rarity within free-text records makes them costly to manually abstract at scale.^13,27,28,29,30^ Although NLP models have been developed to measure this and related constructs,^6,7,12,13,14,31^ the linguistic complexity encountered in documented goals-of-care discussions continues to challenge NLP, and there is ongoing interest in refining NLP approaches to improve performance.^7,14^

In this study, we used deep-learning NLP to measure the primary outcome of EHR-documented goals-of-care discussions in a large pragmatic trial of a communication-priming intervention for hospitalized patients.^32,33^ We evaluated the sensitivity, specificity, and predictive values of our deep-learning NLP model through receiver operating characteristic (ROC) curves and precision-recall analyses and examined the effect of NLP-related misclassification on study power. We concluded by examining the performance, feasibility, and power implications of an NLP-screened human abstraction^34^ approach to measuring the primary outcome of the parent trial.

## Methods

This diagnostic study was conducted to inform selection of an outcome measurement strategy for a pragmatic randomized clinical trial of a communication-priming intervention for hospitalized patients (Project to Improve Communication About Serious Illness—Hospital Study: Pragmatic Trial 1 [PICSI-H Trial 1]).^32,33^ All procedures for this study and the parent clinical trial were approved by the University of Washington institutional review board. Patients were enrolled under an institutional review board–approved waiver of informed consent and Health Insurance Portability and Accountability Act authorization due to minimal risk of the intervention, which was designed to promote best practices. Findings of the current study are reported in accordance with the Transparent Reporting of a Multivariable Prediction Model for Individual Prognosis or Diagnosis (TRIPOD) reporting guideline.^35^

The trial enrolled patients hospitalized at any of 3 study hospitals who either had advanced age (≥80 years) or were aged 55 years or older and had a chronic life-limiting illness as defined by diagnosis codes used by the Dartmouth Atlas Project to study end-of-life care (eTable 1 in Supplement 1).^36,37,38^ Eligible patients were enrolled under a waiver of informed consent and randomized in a 1:1 ratio to usual care or a clinician-facing prompting intervention designed to promote goals-of-care discussions. Clinicians caring for patients in the intervention arm received an e-mailed patient-specific document (“Jumpstart Guide”; eAppendix 1 in Supplement 1) that suggested possible appropriateness of a goals-of-care discussion and provided communication prompts adapted from the VitalTalk communication training model.^39,40^ The primary outcome was EHR documentation of a goals-of-care discussion within 30 days of randomization. Goals-of-care discussions were defined as discussion of the overarching aims of medical care for a patient^15^ and operationalized using a medical record abstraction manual (eAppendix 2 in Supplement 1) adapted from a previous pilot trial.^14,41^ We did not consider stand-alone code status discussions or citations of past advance care planning documents to be goals-of-care discussions. The outcome was measured in all notes written by inpatient and outpatient clinicians (physicians, residents, fellows, subinterns, nurse practitioners, and physician assistants) from the date of randomization to 30 days thereafter.

During planning, the trial was specified to use NLP to measure its primary outcome, with human abstraction as a backup strategy. However, because NLP approaches were developed concurrently with enrollment, the expected degree of NLP-related misclassification was not known at the time the trial began. The trial was initially specified to target a sample size of 2000 participants, which would result in 80% power to detect a difference in proportions of at least 6.2% (assuming a control arm proportion of 0.54 and 2-sided α of 0.05). However, this sample size determination did not consider the potential effect of NLP-related misclassification. Development, training, and testing of NLP models continued throughout enrollment, using data sources gathered from outside the trial.

Between April 23, 2020, and March 26, 2021, the trial enrolled 2512 patients. The prespecified enrollment target of 2000 was exceeded to increase the number of participants with Alzheimer disease and related dementias (ADRD), a prespecified subgroup. Following conclusion of enrollment and prior to unblinding and primary analyses, we froze our NLP program, evaluated NLP performance within a validation sample collected from the trial, and reevaluated the statistical power, human abstraction burden, and pragmatic implications of 3 strategies for measuring the primary outcome: (1) conventional manual abstraction, (2) NLP alone, and (3) NLP-screened human abstraction, in which only EHR passages scored by NLP above a predefined threshold would be reviewed by human abstractors for documented goals-of-care discussions.^34^

### NLP Development and Model Training

We collected a training data set of 4642 EHR notes from 150 participants in a previous pilot trial of a similar patient- and clinician-facing communication-priming intervention at the same study hospitals (Project to Improve Communication About Serious Illness—Pilot Study [PICSI-P]) (eTable 1 in Supplement 1).^41^ Using a codebook adapted from that used to measure the PICSI-P trial outcome (eAppendix 2 in Supplement 1), 5 abstractors (including R.Y.L., J.T.) manually reviewed and coded these 4642 notes for documented goals-of-care discussions using the interface of a qualitative data analysis platform (Dedoose).^42^ Randomization was concealed from abstractors, and instances of disagreement were resolved by consensus.

**Figure 1.**
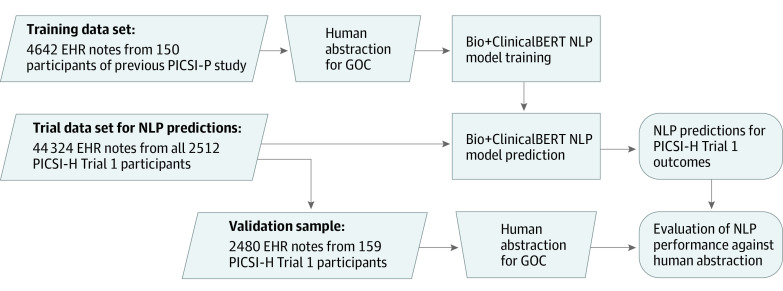
Training, Prediction, and Validation of the Natural Language Processing (NLP) Model EHR indicates electronic health record; GOC, goals of care; PICSI-H Trial 1, Project to Improve Communication About Serious Illness—Hospital Study: Pragmatic Trial (Trial 1)^32,33^; and PICSI-P, Project to Improve Communication About Serious Illness—Pilot Study.^41^

We used the training data set to train Bio+ClinicalBERT, a publicly available and freely distributed deep-learning NLP model, to predict the presence of documented goals-of-care discussions in EHR text (eMethods in Supplement 1).^43,44^ BERT is a deep-learning NLP model developed by Google Research that is pretrained on large quantities of unlabeled text to build a foundation of linguistic information, including how words influence one another’s meaning in context, that can then be applied to a variety of NLP tasks.^45,46,47^ BERT contains a total of 110 million parameters for which values are fitted during pretraining.^45^ Bio+ClinicalBERT is an instance of BERT that was further pretrained on unlabeled biomedical literature and deidentified medical records from the MIMIC III (Medical Information Mart for Intensive Care) database.^43,44,48,49^ To identify documented goals-of-care discussions, we used publicly available software interfaces^50^ to add a classification layer to Bio+ClinicalBERT and trained (“fine-tuned” in BERT parlance) the parameters of the composite model to the training data and its manually abstracted labels for goals-of-care content (Figure 1). As BERT models can only analyze text sequences of limited length (≤512 words or subwords), we used automated algorithms to split each note into component passages compatible with BERT (eMethods in Supplement 1). The resulting fine-tuned model predicts the likelihood of goals-of-care content within any string of candidate text.

### Trial Data Set and Validation Sample

Following conclusion of the PICSI-H Trial 1 outcome assessment period, we used automated database queries to collect all EHR notes authored by attending and trainee physicians, subintern medical students, nurse practitioners, and physician assistants between the date of randomization and 30 days thereafter. This yielded a PICSI-H Trial 1 data set of 44 324 notes from 2512 patients (Figure 1 and eTable 2 in Supplement 1). We randomly selected 159 of these trial participants (eTable 1 in Supplement 1) for manual whole medical record abstraction, oversampling for patients with ADRD (80 of 159 [50%]), yielding a validation sample of 2480 notes (Figure 1 and eTable 2 in Supplement 1). A team of 4 abstractors (including J.T.) manually reviewed and coded all records in this validation sample using the same methods used to label the training data set. Randomization was concealed from abstractors, and instances of disagreement were resolved by consensus.

### Statistical Analysis

#### Evaluating NLP Performance

Following model training and manual abstraction of the validation sample, we used the trained BERT NLP model to predict the probability of documented goals-of-care discussions in all 2.64 million EHR passages (44 324 notes) of the PICSI-H Trial 1 data set. To characterize the expected performance of BERT NLP in the trial data set, we resampled the validation sample to reflect the prevalence of enrolled patients with ADRD (11%) and compared NLP-predicted probabilities for each note and patient against manual abstraction using ROC curves and precision-recall analyses. For all analyses, we report note- and patient-level performance, defining note- and patient-level probability as the maximum predicted probability for all constituent passages and defining the gold-standard label for each note and patient as the union of labels for all constituent passages. Statistical analyses were conducted using Stata/MP, version 17.0 (StataCorp LLC)^51^ with the roctab and prcurve packages.^52^

#### Misclassification-Adjusted Power Calculations

To estimate the detectable risk difference in proportions of patients with documented goals-of-care discussions at a given power in the absence of misclassification, we assumed a control arm prevalence (*p*_1_) of 33.5% based on preliminary data^21,53^ and 2512 patients with 1:1 allocation and calculated the intervention arm proportion (*p*_2_) that would maintain 80% power using a Pearson χ^2^ test with 2-sided α of 0.05.^54,55,56^ To estimate detectable risk difference in the presence of nondifferential misclassification, we substituted sensitivity (*se*)– and specificity (*sp*)–corrected terms representing the observed proportions (*p̂*_1_, *p̂*_2_) into the same power calculation,^10,57^ iterating across values of the true intervention arm proportion (*p*_2_) to identify the value of actual risk difference (*p*_1 _–_ _*p*_2_) at which power to detect a difference between *p̂*_1_ and *p̂*_2_ equaled 80%. The terms for the observed proportions are defined by the following formulae presented by Devine^10^:

*p̂*_1_ = *se* *p̂*_1_ + (1_ _−_ _*sp*)(1_ _−_ _*p̂*_1_)

*p̂*_2_ = *se* *p̂*_2_ + (1 − *sp*)(1 − *p̂*_2_)

To empirically estimate statistical power in the presence of nondifferential misclassification, we performed Monte Carlo simulations constrained to the same sample sizes and control arm prevalence (*p*_1_) over a range of risk differences (*p_2_* – *p_1_*) and values for patient-level sensitivity and specificity. For each of 10 000 replications, we generated true outcomes as binomial variates conditioned on the given values of *n_1_*, *n_2_*, *p_1_*, and *p_2_* and observed outcomes as binomial variates conditioned on the true outcome, sensitivity, and specificity. We then tested for an association between the observed outcome and treatment arm using a χ^2^ test with 2-sided α of 0.05 and reported the proportion of replications that rejected the null hypothesis *(H_0_:* *p̂_1_* *=* *p̂_2_*) as the observed power. We used Bland-Altman analysis to compare observed power in simulations against misclassification-adjusted calculated power.^58^

Power calculations and Monte Carlo simulations were performed using Stata/MP, version 17.0, and further parallelized for performance using the parallel Stata module, version 1.20.0.^59,60^ The source code for misclassification-adjusted power calculations and simulation procedures is provided in eAppendix 3 in Supplement 1. Data were visualized and analyzed using Stata/MP, the blandaltman Stata module,^61^ and Plotly Chart Studio (Plotly). Two-sided *P* < .05 was considered significant.

## Results

Between April 23, 2020, and March 26, 2021, PICSI-H Trial 1 enrolled 2512 patients, whose baseline characteristics are presented in eTable 1 in Supplement 1. The mean (SD) age was 71.7 (10.8) years; 1456 (58%) were female, 1056 (42%) were male, and most had 2 or more chronic life-limiting illness diagnoses (1281 [51%]).

### Abstraction and Composition of Training and Validation Data Sets

In manual abstraction of 4642 EHR notes in the training data set, 340 notes (7%; belonging to 34 of 150 patients [23%]) contained documented goals-of-care discussions (eTable 2 in Supplement 1). The training data set was abstracted using 287 abstractor-hours over a 4-month period.

In manual abstraction of 2840 EHR notes in the validation sample, 268 notes (9%; belonging to 54 of 159 patients [34%]) contained documented goals-of-care discussions (eTable 2 in Supplement 1). Note- and patient-level prevalence of EHR-documented goals-of-care discussions were similar in patients with and without ADRD (note level: 113 of 1248 with ADRD [9%] vs 155 of 1232 without ADRD [13%]; χ^2^ *P* = .81; patient level: 25 of 80 with ADRD [31%] vs 29 of 79 without ADRD [37%]; χ^2^ *P* = .47). Adjusted for ADRD, 12.2% of notes and 36.1% of patients in the validation sample had an EHR-documented goals-of-care discussion. The validation sample was abstracted using 204 abstractor-hours over a 6-month period.

### BERT NLP Performance in the Validation Sample

**Figure 2.**
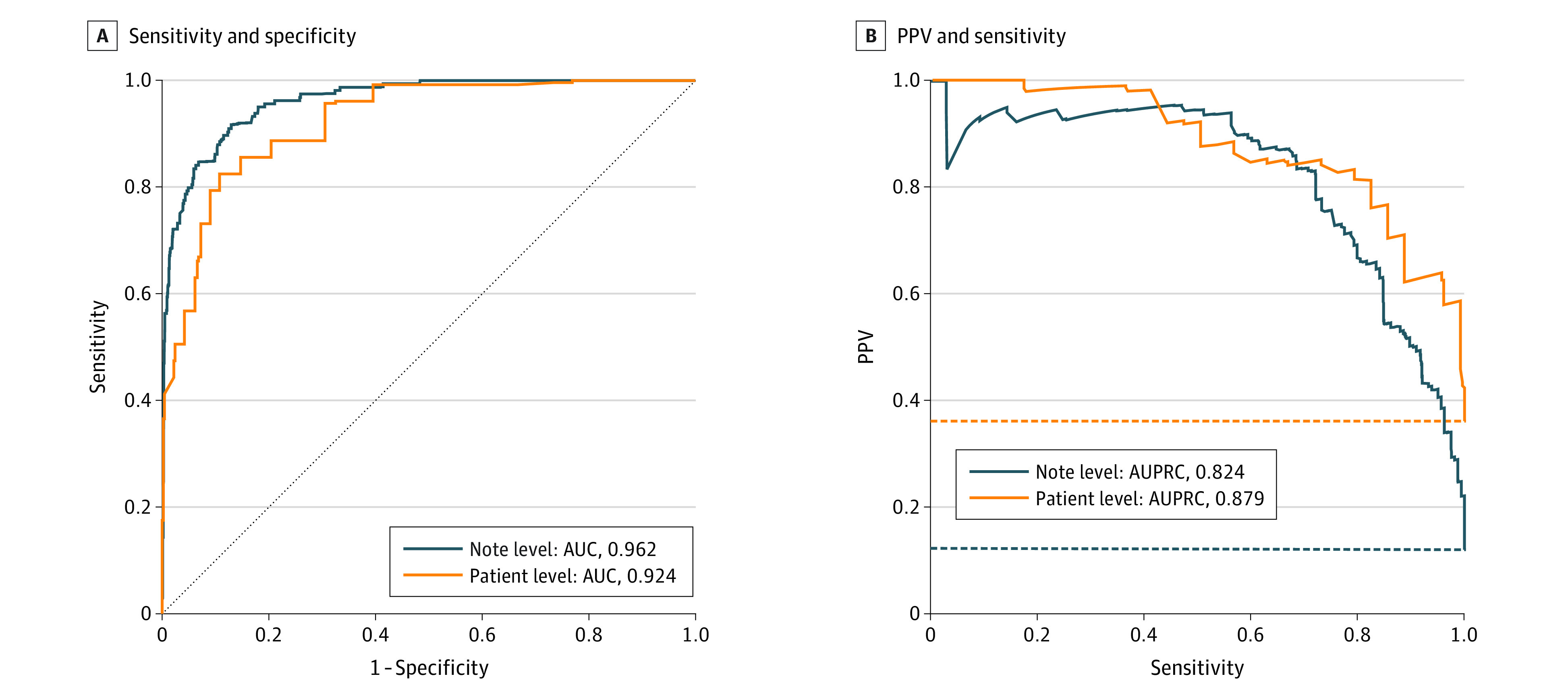
Performance of BERT Natural Language Processing in Classifying 30-Day Documented Goals-of-Care Discussions at Note and Patient Levels in a 159-Patient, 2480-Note Internal Validation Sample Representative values of sensitivity, specificity, positive and negative predictive values, and F1 scores from these curves are presented in the Table. B, Dotted lines indicate nondiscriminating classifiers. AUC indicates area under the receiver operating characteristic curve; AUPRC, area under the precision-recall curve; and PPV, positive predictive value.

In comparing BERT NLP predictions with manual abstraction in the validation sample, BERT NLP demonstrated note- and patient-level areas under the ROC curve of 0.962 and 0.924, respectively (Figure 2). The note- and patient-level areas under the precision-recall curve were 0.824 and 0.879, respectively, and the maximal observed note- and patient-level F_1_ scores were 0.77 and 0.82, respectively. We also report the observed note- and patient-level sensitivity, specificity, positive and negative predictive values, and F_1_ scores at select predefined thresholds (Table).

**Table.** Performance Metrics for BERT NLP in Classifying 30-Day Documented Goals-of-Care Discussions at Note and Patient Levels in a 159-Patient, 2480-Note Internal Validation Sample^a^

Unit of classification	Metric, %				F_1_ score	AUC	AUPRC
	Sensitivity	Specificity	PPV	NPV			
Note level (n = 2480)	70.1	98.1	83.6	95.9	0.76	0.962	0.824
	79.9	94.5	66.9	97.1	0.73		
	89.7	88.1	51.0	98.4	0.65		
Patient level (n = 159)	70.0	92.8	84.5	84.6	0.77	0.924	0.879
	79.4	91.0	83.3	88.6	0.81		
	89.5	69.5	62.3	92.1	0.73		

Abbreviations: AUC, area under the receiver operating characteristic curve; AUPRC, area under the precision-recall curve; NLP, natural language processing; NPV, negative predictive value; PPV, positive predictive value.

^a^ Performance metrics are shown at observed discrimination thresholds with sensitivities closest to prespecified values of 70%, 80%, and 90%.

### Power Estimates With and Without Misclassification

**Figure 3.**
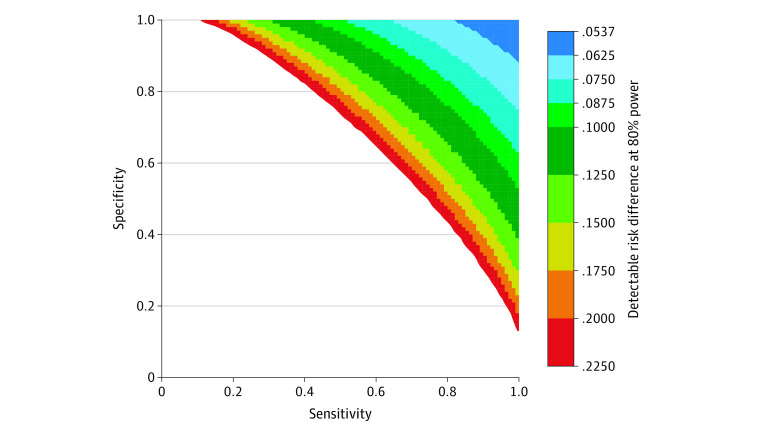
Detectable Risk Difference Over Classifier Performance Assumptions: n1 = 1256; n2 = 1256; p1 = 0.335; power = 0.8; and 2-sided α = .05. An interactive 3-D plot is available at https://chart-studio.plotly.com/~rlee06uw/14/#/.

In conventional power analysis (which assumes no misclassification), we calculated that the trial (N = 2512; 1:1 allocation; assumed *p_1_* of 33.5%) would have 80% power to detect a risk difference of 5.4% with 2-sided α of 0.05. Misclassification-adjusted calculations of detectable risk difference at 80% power across ranges of outcome sensitivity and specificity demonstrated a superlinear increase in detectable risk difference (ie, loss of power) with decreasing sensitivity or specificity (Figure 3). Notably, at this sample size, the detectable risk difference remained under 10% even with substantial misclassification (eg, sensitivity 80%, specificity 80%). Monte Carlo simulations across ranges of sensitivity, specificity, and risk difference demonstrated excellent agreement between calculated and observed (simulated) power in Bland-Altman analysis (eFigure 1 in Supplement 1).

### Comparison of Outcome Measurement Strategies

We evaluated 3 strategies for measuring the primary outcome: manual human abstraction, BERT NLP alone, and BERT NLP–screened human abstraction. The first approach, manual human abstraction, is the de facto gold standard and would power the study to detect a risk difference of 5.4%. Our experience suggested that individual abstractors could perform this abstraction task for up to 3 hours per day before experiencing excessive fatigue. To estimate the number of hours required for complete manual abstraction of the trial data set, we scaled the known abstractor-hours required to collect data for the validation sample to the entire trial data set, yielding an estimate of approximately 3000 abstractor-hours (ie, 67 work weeks for a team of 3 abstractors devoting 3 hours per day to this task; costing $195 000 at a rate of $65 per hour). Based on estimates from the validation sample, constraining abstractors to records from randomization to the first EHR-documented goals-of-care discussion (or 30 days if none was present) would reduce this estimate to approximately 2000 abstractor-hours (ie, 45 work weeks for a team of 3 abstractors, or $130 000 at a rate of $65 per hour).

In the second approach, BERT NLP alone, the primary outcome could be measured with any combination of sensitivity and specificity represented on the ROC curve in Figure 2. Based on the calculated detectable risk differences shown in Figure 3, a BERT NLP model implemented with a discrimination threshold corresponding to a maximal patient-level F_1_ (82.5% sensitivity, 89.2% specificity) would power the study to detect a risk difference of 7.6% compared with 5.4% without misclassification.

**Figure 4.**
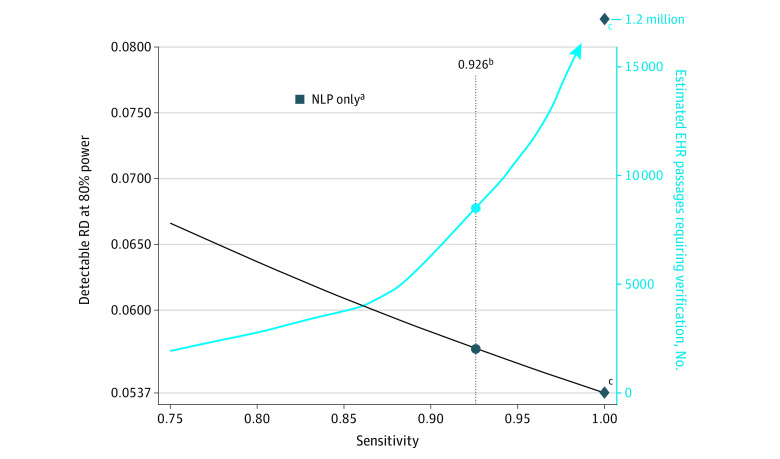
Detectable Risk Difference vs Sensitivity of Natural Language Processing (NLP)–Screened Human Abstraction In considering the use of NLP-screened human abstraction to measure the trial outcome, we evaluated the added utility of human verification of NLP-positive passages compared with NLP alone. Measuring the outcome using NLP alone (square) would have powered the trial to detect a risk difference (RD) of 7.6%. Measuring the outcome using NLP-screened human abstraction would improve power (ie, decrease detectable risk difference) at the cost of the number of passages requiring human verification; this cost increases in a superlinear manner as the screening threshold moves toward higher sensitivity. We ultimately chose to measure the outcome using NLP-screened human abstraction at a sensitivity of 92.6% (circles), which powered the trial to detect a risk difference of 5.7% at the predicted cost of human verification of 8500 NLP-screened electronic health record (EHR) passages. Assumptions: n1 = 1256; n2 = 1256; p1 = 0.335; 2-sided α = .05; power = 0.8. The relationship between power, sensitivity, and detectable risk difference is shown in eFigure 2 in Supplement 1. ^a^Sensitivity and detectable risk difference of BERT NLP alone at the discrimination threshold corresponding to maximal patient-level F_1_ score (82.5% sensitivity, 89.2% specificity). ^b^Sensitivity of NLP-screened human abstraction at the screening threshold selected for the clinical trial. ^c^At 100% sensitivity (perfect classifier; diamonds), the estimated number of NLP-positive EHR passages requiring human abstraction for complete outcomes from date of randomization to first goals-of-care discussion was approximately 1.2 million. Passages contained a median of 52 words each (IQR, 25-101 words).

In the third approach, BERT NLP–screened human abstraction, only EHR passages that were scored by NLP above a predefined threshold would be reviewed by human abstractors for documented goals-of-care discussions.^34^ We used the results shown in Figure 3 to determine the detectable risk difference at 80% power across a range of sensitivities and 100% specificity (Figure 4 and eFigure 2 in Supplement 1) and to estimate patient-level sensitivity and number of EHR passages requiring human verification to achieve complete outcome data from randomization to the first human-confirmed documented goals-of-care discussion (or 30 days if none was present) across a representative range of screening thresholds (Figure 4). All misclassification-adjusted power calculations under these constraints were again consistent with Monte Carlo simulations (eFigure 2 in Supplement 1). Based on these data and the time and resources available, we elected to measure the primary outcome using NLP-screened human abstraction at a screening threshold corresponding to 92.6% estimated patient-level sensitivity. At this threshold, there were 22 187 EHR passages (0.8% of all 2.64 million passages) from 11 287 notes and 1957 patients that screened positive by NLP. Assuming 33.5% control-arm prevalence, abstraction of all NLP-positive passages from randomization to the first human-confirmed documented goals-of-care discussion (or 30 days if none was present) would power the trial to detect a risk difference of 5.7% at 80% power with 2-sided α of 0.05 and was estimated to require manual abstraction of approximately 8500 NLP-screened EHR passages containing a median of 52 words each (IQR, 25-101 words) (Figure 4). Following this decision, 3 research coordinators (including J.T.) adjudicated 7494 EHR passages using 34.3 abstractor-hours over a 3-week period to complete primary outcome measurements for all trial participants from randomization to the first goals-of-care discussion (or 30 days if none was present) at the given screening threshold. The NLP-screened passages were adjudicated by abstractors in a random order, and abstractors were blinded to patient randomization.

## Discussion

In this diagnostic study, we examined the novel use of deep-learning–based NLP to measure a complex outcome from unstructured EHR text in a large pragmatic clinical trial. We also demonstrated and validated the use of statistical methods to quantitatively assess the effects of NLP-related misclassification on study power at a given sample size.

Natural language processing–screened human abstraction represents an efficient and useful approach for measuring EHR outcomes in large pragmatic studies and is increasingly used to measure palliative care outcomes similar to the one we examined.^34,62,63,64,65^ In the 2512-patient PICSI-H Trial 1, measuring the primary outcome using conventional manual abstraction would have required thousands of abstractor-hours, which is both costly and time consuming. In contrast, NLP-screened human abstraction allowed investigators to make up-front investments in developing NLP and collecting training and validation data and then measure the primary outcome during a smaller number of abstractor-hours with acceptable losses in sensitivity and power. Misclassification-adjusted interim power analyses allowed investigators to select a strategy for measuring the primary outcome that best balanced statistical power with abstractor time and resources.

Researchers considering NLP or NLP-aided approaches to measuring outcomes should consider the effects of the outcome measurement strategy on the statistical power, costs, and validity of the trial. In this study, we demonstrated 2 simple methods for researchers to perform misclassification-adjusted power calculations, and we encourage the uptake of this approach in the design of future trials.^9,10^ We also demonstrated the utility of NLP-screened human abstraction^34^ to measure trial outcomes from the EHR. It should be noted that in implementing NLP-screened human abstraction of EHR passages, both the expected sensitivity and the number of EHR passages that require human verification are functions of the selected NLP screening threshold in a given data set; an analysis such as that shown in Figure 4 may help investigators select the most appropriate screening threshold for their study. Notably, at a given threshold score, the patient-level sensitivity of NLP-screened human abstraction can be lower than that of NLP alone due to the potential presence of documented goals-of-care discussions scoring beneath the screening threshold in patients for whom all NLP-positive passages are false-positive. Because of this, studies that use NLP-screened human abstraction of EHR passages must estimate patient-level sensitivity at a given screening threshold in the sample population either by statistical methods or by testing within validation data sampled at the patient level.

Perhaps the most conspicuous limitation of using NLP to measure clinical research outcomes is the investment required to implement NLP. Although this investment may be minimal for outcomes that are easily detected using rule-based NLP, identifying more complex constructs such as the one investigated here may require substantial software development and acquisition of training and validation data. Our research group has spent more than 500 developer-hours implementing this NLP model, in addition to 491 abstractor-hours spent collecting training and validation data. We anticipate that the cost of implementing high-performing pretrained NLP models will decrease as the field matures. Additionally, many NLP development costs are fixed with respect to the number of study participants, and many aspects of NLP development are transferrable to other studies and research questions. Although our BERT NLP model is not yet portable due to the privacy risks associated with training on identifiable protected health information,^66^ we believe it represents a first step toward the development of a publicly available, validated, and generalizable BERT NLP model for identifying EHR-documented goals-of-care discussions.

### Limitations

Our study has several important limitations. First, our model was trained and validated on data from a single health system, and its performance may not generalize to other systems. Future external validation and efforts to improve explainability of deep learning models^67,68^ will be important to developing models that generalize across health systems. Second, our model was validated within a relatively small sample of 159 patients, which limits our assessment of generalizability outside the validation sample. Although the performance observed in the validation sample was comparable with that observed in within-training-set leave-one-group-out cross-validation procedures, these estimates have the same limitation of small sample size. Given the high cost of abstracting this outcome per patient hospitalization, future validation of NLP models measuring this outcome should consider alternative sampling strategies or statistical methods that account for incomplete reference data.^69,70^ Third, the limited diversity of the validation sample limited our ability to examine potential patient-level biases in the resulting model.^71^ It is possible that our model performs differently across variables such as race, ethnicity, gender, or other clinical and socioecological patient characteristics. While such differential performance is less likely to bias the findings of a randomized clinical trial (in which predictors are randomly distributed between groups), observational studies of NLP-measured outcomes must consider differential misclassification over exposures as a potential source of bias. This issue is particularly salient for palliative care researchers due to the known role of race, ethnicity, and racism in palliative care disparities.^53,72^ As clinical NLP matures toward developing clinical predictive models, it will be imperative to thoroughly validate model performance and generalizability over diverse socioecological groups to avoid perpetuating health disparities. Fourth, although NLP-screened human abstraction may facilitate larger sample sizes than manual data collection, the scalability of this approach is inherently limited by variable costs of human abstraction that increase with sample size. Fifth, our conclusions for researchers are not applicable to all clinical outcomes. Although the outcome we examined is linguistically complex, other outcomes of interest may be even more difficult to classify using EHR text, and the potential of NLP to screen for or measure such outcomes may be limited. Sixth, we did not examine the effects of NLP-related outcome misclassification on estimates of exposure-outcome associations. Recent advances in statistical methods that account for outcome misclassification have shown promise for enhancing the validity of such estimates,^8,9,73,74^ and we believe clinical researchers who use NLP to measure outcomes should consider adopting such methods in their analyses.

## Conclusions

In this diagnostic study evaluating the use of deep-learning NLP to measure EHR-documented goals-of-care discussions, we measured the primary outcome of a large pragmatic clinical trial using NLP-screened human abstraction with acceptable sensitivity and substantial savings in abstractor-hours. Our experience demonstrated that NLP may facilitate clinical research studies that would otherwise be infeasible due to the costs of manual medical record abstraction. Misclassification-adjusted power calculations quantified power loss from NLP-related misclassification, suggesting that incorporation of this approach into the design of future studies that use NLP to measure outcomes would be beneficial.

## Notes

Supplement 1. **eTable 1.** Patient Characteristics by Note Corpus **eTable 2.** Characteristics of Electronic Health Record Note Corpora **eFigure 1.** Comparison of Calculated vs Observed (Simulated) Power Over Ranges of Sensitivity, Specificity, and Risk Difference **eFigure 2.** Power vs Detectable Risk Difference vs Sensitivity of NLP-screened Human Abstraction
eMethods.
**eAppendix 1.** Examples of Clinician-facing Communication-priming Intervention Forms (Jumpstart Guide) From Clinical Trial **eAppendix 2.** Chart Abstractor Codebook for the Project to Improve Communication in Serious Illness (PICSI) Trial Series **eAppendix 3.** Stata Source Code for Study Power Calculation and Simulation Procedures
eReferences

Supplement 2.
Data Sharing Statement
