# Supplementary material for: Assessment of Natural Language Processing of Electronic Health Records to Measure Goals-of-Care Discussions as a Clinical Trial Outcome
Source: JAMA Netw Open. 2023 Mar 2;6(3):e231204. doi: 10.1001/jamanetworkopen.2023.1204 (PMC9982698; doi:10.1001/jamanetworkopen.2023.1204)
Supplement: Supplement 1. — eTable 1. Patient Characteristics by Note Corpus eTable 2. Characteristics of Electronic Health Record Note Corpora eFigure 1. Comparison of Calculated vs Observed (Simulated) Power Over Ranges of Sensitivity, Specificity, and Risk Difference eFigure 2. Power vs Detectable Risk Difference vs Sensitivity of NLP-screened Human Abstraction eMethods. eAppendix 1. Examples of Clinician-facing Communication-priming Intervention Forms (Jumpstart Guide) From Clinical Trial eAppendix 2. Chart Abstractor Codebook for the Project to Improve Communication in Serious Illness (PICSI) Trial Series eAppendix 3. Stata Source Code for Study Power Calculation and Simulation Procedures eReferences [file jamanetwopen-e231204-s001.pdf]

# Supplementary Online Content

Lee RY, Kross EK, Torrence J, et al. Assessment of natural language processing of electronic health records to measure goals-of-care discussions as a clinical trial outcome. *JAMA Netw Open*. 2023;6(3):e231204. doi:10.1001/jamanetworkopen.2023.1204

**eTable 1.** Patient Characteristics by Note Corpus
**eTable 2.** Characteristics of Electronic Health Record Note Corpora
**eFigure 1.** Comparison of Calculated vs Observed (Simulated) Power Over Ranges of Sensitivity, Specificity, and Risk Difference
**eFigure 2.** Power vs Detectable Risk Difference vs Sensitivity of NLP-screened Human Abstraction
**eMethods**
**eAppendix 1.** Examples of Clinician-facing Communication-priming Intervention Forms (Jumpstart Guide) From Clinical Trial
**eAppendix 2.** Chart Abstractor Codebook for the Project to Improve Communication in Serious Illness (PICSI) Trial Series
**eAppendix 3.** Stata Source Code for Study Power Calculation and Simulation Procedures
**eReferences**

This supplementary material has been provided by the authors to give readers additional information about their work.

© 2023 Lee RY et al. *JAMA Network Open*.

**eTable 1.** Patient Characteristics by Note Corpus

| Patient characteristic | Training dataset (PICSI-P Trial) [101] N = 150 | Trial dataset for NLP predictions (PICSI-H Trial 1) [102,103] N = 2,512 | Validation sample of trial dataset [a] n = 159 |
|---|---|---|---|
| Age, years, mean (SD) | 59.2 (13.6) | 71.7 (10.8) | 72.6 (10.6) |
| Sex, n (%) | | | |
| Female | 66 (44) | 1,456 (58) | 66 (42) |
| Male | 84 (56) | 1,056 (42) | 93 (58) |
| Race, n (%) | | | |
| Asian | 2 (1) | 292 (12) | 24 (15) |
| Black | 19 (13) | 316 (13) | 36 (23) |
| Native American | 2 (1) | 45 (2) | 3 (2) |
| Pacific Islander | 0 (0) | 13 (0.5) | 1 (1) |
| White | 116 (77) | 1,768 (70) | 94 (59) |
| Mixed | 10 (7) | | |
| Unknown | 1 (1) | 78 (3) | 1 (1) |
| Ethnicity, n (%) | | | |
| Hispanic | 9 (6) | 150 (6) | 9 (6) |
| Non-Hispanic | 107 (71) | 2,347 (93) | 149 (94) |
| Unknown | 34 (23) | 15 (1) | 1 (1) |
| Eligibility criteria (not mutually exclusive) | | | |
| Age ≥ 80 years | 9 (6) | 685 (27) | 53 (33) |
| Age ≥ 65 years with frailty [b] | 11 (7) | N/A | N/A |
| Chronic life-limiting illness diagnoses [c] | | | |
| Cancer with poor prognosis | 25 (17) | 596 (24) | 30 (19) |
| Chronic lung disease | 46 (31) | 680 (27) | 46 (29) |
| Coronary artery disease | 43 (29) | 866 (34) | 71 (45) |
| Congestive heart failure | 52 (35) | 698 (28) | 67 (42) |
| Peripheral vascular disease | 21 (14) | 538 (21) | 42 (26) |
| Severe chronic liver disease | 25 (17) | 315 (13) | 20 (13) |
| Diabetes with end-organ damage | 21 (14) | 386 (15) | 32 (20) |
| Moderate-to-severe chronic kidney disease | 56 (37) | 627 (25) | 42 (26) |
| Alzheimer disease and related dementias | 6 (4) | 280 (11) | 80 (50) |

*(continued on next page)*

© 2023 Lee RY et al. *JAMA Network Open*.

| Patient characteristic | Training dataset (PICSI-P Trial) [101]<br><br>N = 150 | Trial dataset for NLP predictions (PICSI-H Trial 1) [102,103]<br>N = 2,512 | Validation sample of trial dataset [a]<br><br>n = 159 |
|---|---|---|---|
| No. chronic life-limiting illness diagnoses | | | |
| 0 | 10 (7) | 326 (13) | 19 (12) |
| 1 | 64 (43) | 905 (36) | 36 (23) |
| 2 | 24 (16) | 485 (19) | 25 (16) |
| 3 | 50 (33) | 361 (14) | 29 (18) |
| ≥ 4 | 64 (43) | 435 (17) | 50 (31) |

[a] Patients in the validation sample are a subset of the patients in the trial dataset for NLP predictions (PICSI-H Trial 1).
[b] In the PICSI-P trial, frailty was defined by serum albumin ≤ 3.0 within 48 hours of admission[104-107] and documented weight loss of 10 or more pounds over the preceding year.[108,109] Patients 65 years of age or older with frailty were eligible for enrollment in PICSI-P. Frailty was not used as an eligibility criterion in PICSI-H Trial 1.
[c] Chronic life-limiting illness diagnoses are not mutually exclusive.

Abbreviations: PICSI-P, Project to Improve Communication About Serious Illness—Pilot Trial;[101] PICSI-H Trial 1, Project to Improve Communication About Serious Illness—Hospital Trial 1;[102,103] SD, standard deviation; N/A, not applicable.

© 2023 Lee RY et al. *JAMA Network Open*.

**eTable 2.** Characteristics of Electronic Health Record Note Corpora

| Characteristic | Training dataset (PICSI-P Trial) [101] | Trial dataset for NLP predictions (PICSI-H Trial 1) [102,103] | Validation sample of trial dataset [a] |
|---|---|---|---|
| Number of patients represented | 150 | 2,512 | 159 |
| No. (%) patients with GOCD by manual abstraction | 34 (23) | N/A [b] | 54 (34) |
| No. notes per patient, median (IQR) | 19 (11-29) | 12 (7-22) | 10 (6-19) |
| Number of EHR notes | 4,642 | 44,324 | 2,480 |
| No. (%) notes with GOCD by manual abstraction | 340 (7) | N/A [b] | 268 (11) |
| Note length, words, median (IQR) | 1,070 (689-1,500) | 1,039 (668-1,468) | 1,053 (663-1,537) |
| Note length, BERT tokens, median (IQR) | 2,377 (1,578-3,202) | 2,027 (1,292-2,838) | 2,027 (1,269-2,931) |
| No. unique BERT tokens per note, median (IQR) | 690 (519-848) | 639 (476-800) | 646 (467-829) |
| Number of BERT segments (≤ 512 tokens in length) | 201,394 | 2,644,387 | 103,566 |
| No. (%) BERT segments with GOCD by manual abstraction | 446 (0.2) | N/A [b] | 519 (0.5) |
| Segment length, words, median (IQR) | 9 (3-30) | 6 (6-19) | 11 (4-34) |
| Segment length, BERT tokens, median (IQR) | 14 (4-42) | 14 (9-36) | 23 (10-70) |
| No. unique BERT tokens per segment, median (IQR) | 11 (4-29) | 14 (9-29) | 20 (10-48) |

[a] Notes in the validation sample are a subset of notes in the trial dataset for NLP predictions (PICSI-H Trial 1).
[b] Manual abstraction was not performed on the entirety of the trial dataset.

Abbreviations: PICSI-P, Project to Improve Communication About Serious Illness—Pilot Trial;[101] PICSI-H Trial 1, Project to Improve Communication About Serious Illness—Hospital Trial 1;[102,103] GOCD, goals-of-care discussion; N/A, not applicable; EHR, electronic health record; IQR, interquartile range; BERT, Bidirectional Encoder Representations from Transformers.

© 2023 Lee RY et al. *JAMA Network Open*.

**eFigure 1.** Comparison of calculated vs observed (simulated) power over ranges of sensitivity, specificity, and risk difference [a]

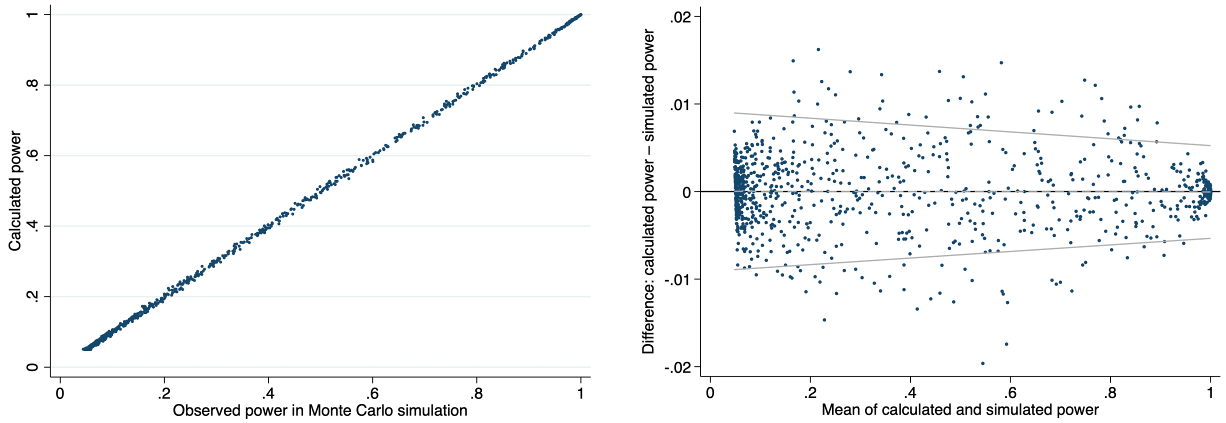


[a] Assumptions: $n_1$ 1256, $n_2$ 1256, $p_1$ 0.335, two-sided $\alpha$ 0.05. Ranges for randomly-sampled assumed values: risk difference, [0.0025, 0.2225]; sensitivity, [0.01, 1.0]; specificity, [0.01, 1.0]. Monte Carlo simulations were run over 10,000 replications. Comparison by Bland-Altman analysis: Mean difference −0.000004 (95%CI −0.003, 0.003; p=0.98); 95% limits of agreement −0.008, 0.008.

© 2023 Lee RY et al. *JAMA Network Open*.

**eFigure 2.** Power vs Detectable Risk Difference vs Sensitivity of NLP-screened Human Abstraction [a]

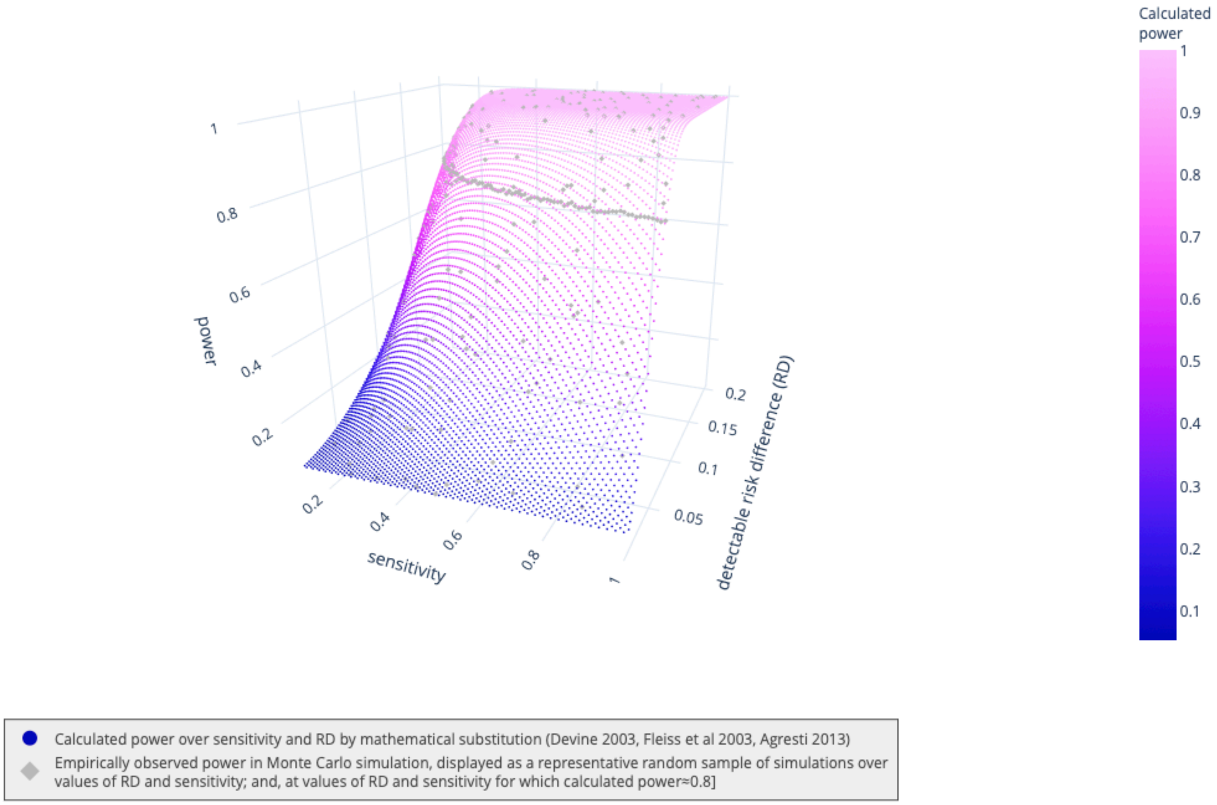


[a] Assumptions: $n_1$ 1256, $n_2$ 1256, $p_1$ 0.335, two-sided $\alpha$ 0.05, specificity 1. Empirically observed power in Monte Carlo simulation was consistent with calculated power by mathematical substitution for all observations. The intersection of this surface and power=0.8 at sensitivity ≥ 0.75 is shown in **Figure 4**. Interactive 3-dimensional plot available at <https://chart-studio.plotly.com/~rlee06uw/1>.

Abbreviations: NLP, natural language processing; RD, risk difference.

© 2023 Lee RY et al. *JAMA Network Open*.

**eMethods**

NLP passage splitting and tokenization.

Textual records from each dataset were rendered as unformatted plain-text, and split into words and subwords from BioClinicalBERT's pre-defined vocabulary.[110,111] These words and subwords (e.g. stems, prefixes, suffixes, or characters) are termed "tokens", and they represent the smallest unit of analysis of BERT models. We further split tokenized notes into non-overlapping passages of ≤ 512 tokens each (BERT's upper-limit for sequence length) using common delimiting whitespace patterns. Passages exceeding this 512-token limit without intervening delimiting whitespace patterns were split evenly into non-overlapping subpassages of ≤ 512 tokens each. These BERT-compatible subpassages constituted the text sequences analyzed by BERT during both model training and prediction. During model training and validation, labels assigned to spans of text by human abstractors were applied to all BERT passages that intersected with these spans.

The training dataset of 4,642 notes from 150 patients was split into 201,394 non-overlapping passages of ≤ 512 tokens each; the trial dataset for NLP prediction of 44,324 notes from 2,512 patients was split into 2.64 million passages; and, the validation sample of the trial dataset of 2,480 notes from 159 patients was split into 103,566 passages.

NLP model architecture

Our NLP model is a fine-tuned instance of BioClinicalBERT, a publicly-available deep-learning NLP model.[110,112] $\text{BERT}_{\text{BASE}}$ (Bidirectional Encoder Representations from Transformers—base model) is a Transformer-based 12-layer 768-hidden 12-head NLP model developed by Google Research that was released as free software in 2018 as case-sensitive ($\text{BERT}_{\text{BASE-Cased}}$) and case-insensitive ($\text{BERT}_{\text{BASE-Uncased}}$) pre-trained models.[111,113] $\text{BERT}_{\text{BASE}}$ models were pre-trained to predict masked words within large corpuses of unlabeled English texts (i.e. the full texts of English Wikipedia and BookCorpus [a dataset of 11,000 unpublished books]), and its learning mechanism analyzes the relationship of each token to other tokens encountered in its vicinity in the pre-training data.[111,113] In a given text sequence, each token is represented by a multidimensional vector in the input layer of the model, and each subsequent layer performs a set of transformations on the output of the preceding layer to generate context-specific representations of tokens in a neural-network-like model.[114] The ~110 million parameters in the $\text{BERT}_{\text{BASE}}$ model are fitted during pre-training.[111] $\text{BioBERT}_{\text{(Base v1.0 + PubMed 200K + PMC 270K)}}$ is an instance of $\text{BERT}_{\text{BASE-Cased}}$ that was further pre-trained on 200,000 PubMed abstracts and 270,000 PubMed Central manuscripts to adapt it to the biomedical domain;[115,116] and, BioClinicalBERT is an instance of the above version of BioBERT that was further pre-trained on 2.1 million EHR notes from the MIMIC III database.[110,112] All three of these models share the $\text{BERT}_{\text{BASE}}$ architecture, and share a 30,000-token vocabulary that was defined during the pre-training of $\text{BERT}_{\text{BASE}}$. Any

© 2023 Lee RY et al. *JAMA Network Open*.

encountered word that is not represented in the vocabulary is repeatedly parsed into component subwords until all constituent tokens are represented in the vocabulary. All BERT pre-trainings are self-supervised, as masked words are treated as their own labels for prediction; they involve no human labeling of the pre-training texts.

© 2023 Lee RY et al. *JAMA Network Open*.

**eAppendix 1.** Examples of clinician-facing Communication-priming Intervention Forms (Jumpstart Guide) From Clinical Trial

## (a) Paper version

UW Medicine

Date Created: mm/dd/yy

### Jumpstart Guide

**Your patient may benefit from a goals of care talk.**

We have chosen your patient based on a diagnosis of one or more chronic illnesses.
Please treat this guide as CONFIDENTIAL.

**Your patient: John Doe, MRN: H1234567**

| | | |
|---|---|---|
| Code Status | Full code | 10/15/2019 |
| Advance directive | YES | 12/15/2018 |
| DPOA health care | NO | |
| POLST | NO | |

**1. Give yourself 5-10 minutes.** The conversation does not have to be long.

**2. Introduce the talk as a routine part of care.** Some patients are reluctant— don't start with death or CPR.

"I want to know what's important to you so that we provide the best care to fit your goals. Is that okay?"

**3. Pick the best topics for your patient.** You don't have to do them all.

| ***Topics*** | ***Words to try*** |
|---|---|
| Understanding | "What have other doctors told you about how serious your illness is and what to expect?" |
| Acceptable states | "What abilities are so important to you that you can't imagine living without them?" |
| Values | "If you were to get sicker, what would be most important to you?" |

**4. Document a short note.** A brief summary and a quote (a few of the patient's words) are enough. Your colleagues will appreciate it.

[study name] Study / [phone #] / [study team e-mail]

## (b) Electronic version

### Jumpstart Guide: A UW Medicine Program

**Your patient may benefit from a goals of care talk.**

We have chosen your patient based on a diagnosis of one or more chronic illnesses.
Please treat the PHI on this guide as CONFIDENTIAL - okay to share with team.

**Your patient: John Doe, MRN: H1234567**

| | | |
|---|---|---|
| Code Status | Full code | 10/15/2019 |
| Advance directive | YES | 12/15/2018 |
| DPOA health care | NO | |
| POLST | NO | |

**1. Give yourself 5-10 minutes.** The conversation does not have to be long.

**2. Introduce the talk as a routine part of care.** Some patients are reluctant — don't start with death or CPR.

"I want to know what's important to you so that we provide the best care to fit your goals. Is that okay?"

**3. Pick the best topics for your patient.** You don't have to do them all.

| ***Topics*** | ***Words to try*** |
|---|---|
| Understanding | "What have other doctors told you about how serious your illness is and what to expect?" |
| Acceptable states | "What abilities are so important to you that you can't imagine living without them?" |
| Values | "If you were to get sicker, what would be most important to you?" |

**4. Document a short note.** A brief summary and a quote (a few of the patient's words) are enough. Your colleagues will appreciate it.

**Optional Feedback**

Select an option below to send us feedback on this message.

Will Definitely Do | Will Do If Time Allows | Maybe, Will Consider

Not Appropriate | Already Done | Other

*Brought to you by UW Medicine and the* [study name] *Research Team. To reach us, call* [phone #] *or email* [study staff contact].

*Date created: mm/dd/yy*

© 2023 Lee RY et al. *JAMA Network Open*.

**eAppendix 2.** Chart Abstractor Codebook for the Project to Improve Communication in Serious Illness (PICSI) Trial Series

# Chart Abstractor Codebook
# for
# Project to Improve Communication in Serious Illness (PICSI) Trial Series

**Codebook authors:** Robert Y. Lee, MD, MS; Kelson Okimoto, MSW, RHIA, LSWAIC; Janaki M. Torrence, MS, CCC-SLP; Patsy D. Treece, RN, MN; Elizabeth L. Nielsen, MPH; Joanna Heywood, BA; Sudiptho R. Paul, MS; Erin K. Kross, MD; Ruth A. Engelberg, PhD; J. Randall Curtis, MD, MPH.  All authors are affiliated with the Cambia Palliative Care Center of Excellence at UW Medicine, University of Washington School of Medicine, Seattle, WA.

**Last revised:** March 23, 2021

**Overview and revision history:** This codebook is the product of a collaborative effort by its authors to identify and operationalize working definitions for content domains commonly encountered in clinician documentation of serious illness communication in the UW Medicine electronic health record. Following a literature-informed drafting of a preliminary codebook, the authors engaged in an iterative process of descriptive coding, deliberation, and refinement of coding definitions and examples to arrive at the codebook presented. This process was informed by qualitative abstraction of free-text electronic health records belonging to a broad array of outpatients and inpatients with serious illness. The team met weekly over a 4-month period in 2020 to arrive at a consensus for coding instructions, definitions, and examples, and made further refinements based on application of the codebook to real-world electronic health record data. A subsequent major revision in March 2021 using a similar iterative approach improved reproducibility of abstraction for clinical notes considered in isolation.

**Acknowledgements:** This work was supported by the National Palliative Care Research Center, the National Institute on Aging (AG062441), the National Heart, Lung, and Blood Institute (HL137940), and the Cambia Health Foundation. The PICSI trials are registered at ClinicalTrials.gov (NCT03746392, NCT04281784, NCT04283994).

© 2023 Lee RY et al. *JAMA Network Open*.

# Identifying Goals-of-Care Discussions in the EHR

## What are "Goals of Care"?

**Goals of care (GOC)** is a term that refers to "the overarching aims of medical care for a patient." These overarching aims might include some combination of priorities surrounding life extension, avoiding disability, relief from pain or discomfort, or other goals. Ideally, goals of care should be "informed by patients' underlying **values and priorities**, established within the existing clinical context, and used to guide **decisions about the use of, or limitation(s) on, specific medical interventions**." [Secunda et al, JGIM 2019]

Conversations between patients, families, and clinicians about goals of care are common among patients with serious illness. These conversations are referred to as **goals of care discussions**. These discussions may be prompted by a chronic, life-limiting medical condition; by a significant clinical event, such as a hospitalization; and/or, by the development of a new acute illness for which a decision regarding specific treatments must be made at that time.

**A GOC discussion may include discussion of values, goals, and treatment preferences/limitations.** While the ideal GOC discussion always includes all three elements, real-life documentation often does not include all three elements.

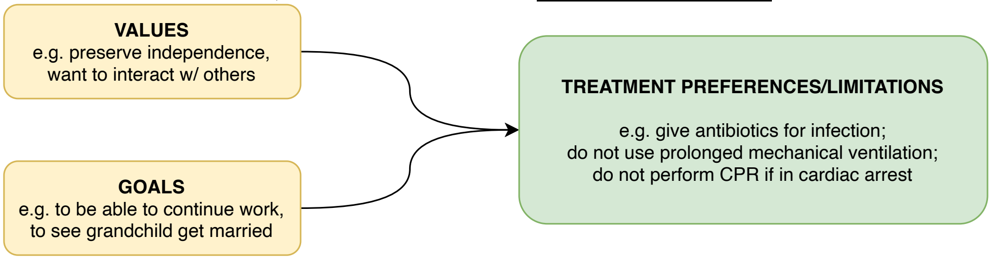


## Project Objective

Our goal is to measure **documentation of conversations with patients and families about goals of care.** As the exact definition of what constitutes a GOC discussion may change from study to study depending on the research question being asked, this codebook will define several different domains of documentation of GOC that will serve future studies measuring the timing, frequency, and nature of GOC discussions, and their documentation in the EHR.

## Related Terminology

**Advance care planning (ACP):** "A process that supports adults in understanding and sharing their personal values, life goals, and preferences regarding future medical care. The goal of advance care planning is to help ensure that people receive medical care that is consistent with their values, goals and preferences during serious and chronic illness." [Sudore et al]

**ACP forms/documents:** Specific (usually paper) forms used to codify and document the results of advance care planning, often by translating them into specific directives or treatment limitations. ACP forms include advance directives (AD), healthcare directives, living wills for healthcare (LW, LWHC), healthcare DPOA forms, and POLST forms.

**Advance directive (AD):** This is a term that refers to several specific types of ACP documents, including healthcare directives, living wills for healthcare (LW, LWHC), and healthcare DPOA forms.

**DNR** or **DNAR:** Do not resuscitate; do not attempt resuscitation (synonymous). Translation: "In the event of cardiac arrest, allow natural death and do not perform CPR."

**DNI:** Do not intubate. **Intubation** refers to insertion of a breathing tube and initiation of invasive mechanical ventilation.

**Code status:** An in-hospital physician order specifying whether the patient should receive CPR in event of a cardiac arrest (DNR vs. full code), and also whether the patient should receive intubation and mechanical ventilation in the event of respiratory failure (DNI vs. OK-to-intubate). Full code presumes OK-to-intubate. ("To code" means to have one's heart stop [i.e. cardiac arrest].)

**DPOA:** Durable power of attorney. In medicine, this usually refers to one's healthcare power of attorney (**HCDPOA** or **DPOAHC**). Individual(s) designated by the patient as healthcare DPOA have statutory authority to make healthcare decisions for the patient if/when the patient becomes incapacitated. In Washington State, healthcare DPOA is documented by a notarized form.

**Hospice:** A palliative, comfort-measures-only (CMO) approach to caring for patients with terminal illness. In the US, this usually refers to a specific government-run Medicare program that provides payment for this type of care to eligible enrollees.

**LNOK:** Legal next-of-kin. This refers to the specific surrogate decision-maker(s) that have been granted statutory authority in Washington State to make healthcare decisions on behalf of an incapacitated patient (RCW 7.70.065).

**POLST:** Physician Orders for Life-Sustaining Treatment. This is a special type of ACP form that contains portable medical orders to guide emergency treatment (see wsma.org/polst and polst.org). It includes the option for a portable DNR order, and other orders.

© 2023 Lee RY et al. *JAMA Network Open*.

# General Principles and Practices

## Coding Practices

When coding, define boundaries of passages at a "macro" level. Coded passages should be comprised of sentences or clauses at the smallest, up to multiple paragraphs at the largest (e.g. a long GOC discussion). Try to capture enough text to give a reader context as to why it is coded the way you coded it. In the case of non-prose text (such as a checklist, or a bulleted list of treatment decisions), only include in your excerpt those bulleted items that directly pertain to the codes you are using. Here is an example:

> *All highlighted text (any color): coded as GOCD*
>
> *Prognosis* *GOCD-Values/Goals and GOCD-Treatments* *GOCD-Values/Goals*

*# GOC*
*Very poor prognosis. Extensive family meeting on 3/15/22 with his uncle and aunt; all agree that comfort should be priority, and that ongoing long-term mech ventilation not within GOC.*
*-- uptitrating fentanyl gtt for air hunger to 200 mcg/hr, with plan for terminal extubation*
*-- weaning off of propofol and dexmedetomidine*
*-- d/c antibiotics*
*-- family wants a priest to visit as the patient was a devout Catholic*
*-- appreciate palliative care consult*
*-- family has expressed goal of pt dying at home; may be possible depending on post-extubation trajectory, although symptom management will be challenging; will explore feasibility depending on nursing and respiratory assessment and will explore logistics with SW*
*-- continuing steroids for now as it seems of low harm although may stop these going forward too*

## Coding Hierarchy

Codes in this codebook have one of three purposes:

1. Codes documenting GOC discussions and related information → Section A
2. Codes to help us develop future instruments to measure quality of documentation of GOC discussions → Section A, sub-codes of GOCD
3. Codes to identify text that might be mistaken for GOC content → Section B

Because some of these goals are subservient to others, we have defined a coding hierarchy for when to apply certain codes, and when certain codes may be ignored.
**Please follow the coding hierarchy (next page) when coding.**

## What if I'm not sure if a given code applies?

There is a special code in Dedoose called FLAG for review that can flag a given coding for further review. We encourage you to always make your best effort coding (i.e. apply the codes as best as you see fit), then apply the code FLAG for review to the passages in question and write a memo containing your questions.

© 2023 Lee RY et al. *JAMA Network Open*.

Ideally, the number of flagged codes should decrease over time as you become more familiar with the constructs that the codebook is trying to measure.

## GOCD and its sub-codes

GOCD is the most all-encompassing code. When using it, please tag the *entirety* of the GOC discussion. (See above under “Coding practices” for more detail on how to code longer passages.) Subcodes of GOCD need only be applied within passages that are coded GOCD. Content representing a subcode that occurs *outside of* a GOC discussion need not be coded.
Subcodes of GOCD should be reserved for instances when the defined concept “jumps out at you with clarity.” Some GOC discussions may not contain any such passages.

© 2023 Lee RY et al. *JAMA Network Open*.

# Coding Flowchart

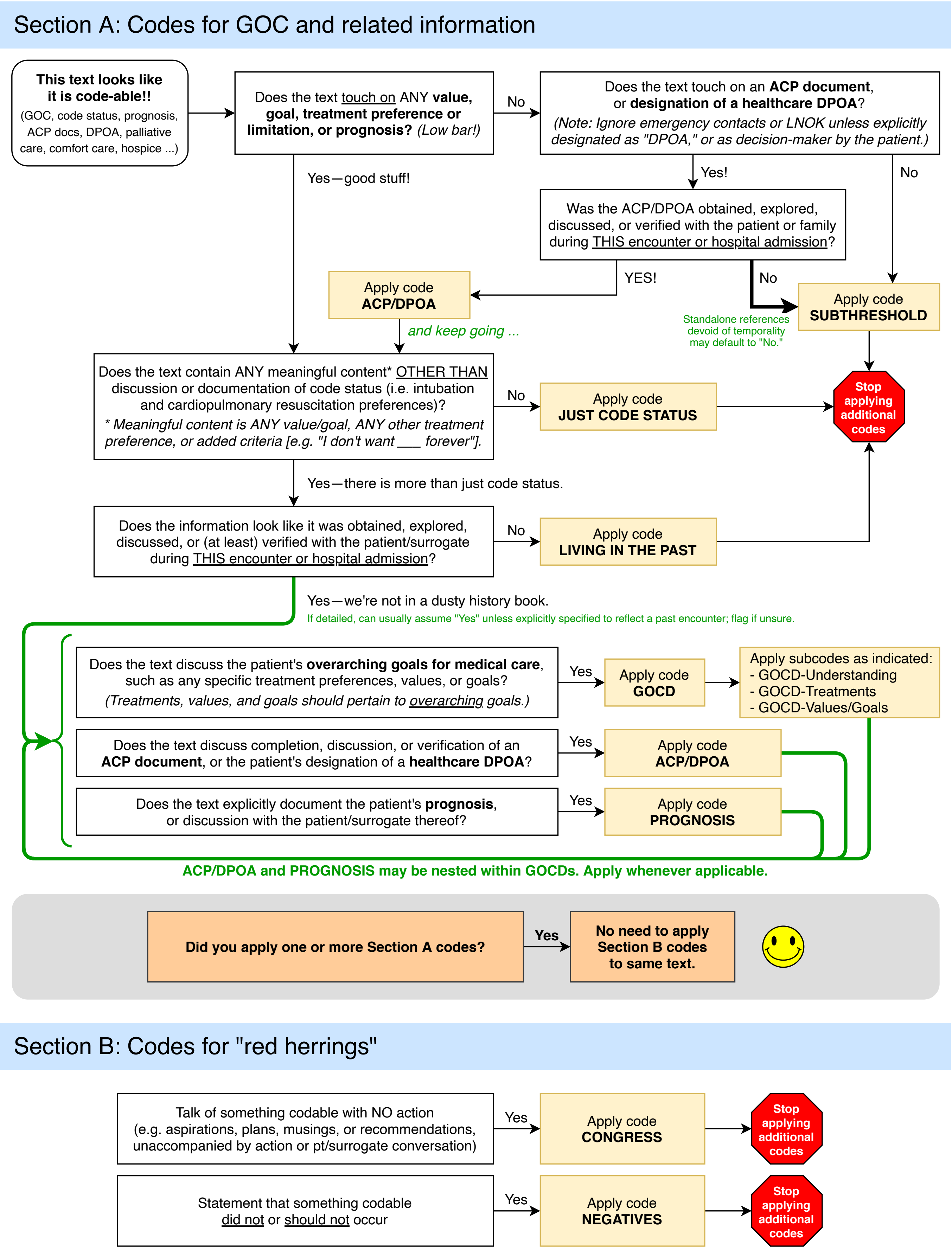


© 2023 Lee RY et al. *JAMA Network Open*.

## **Section A:** Codes for GOC and related information

Codes in this section fit into 3 broad categories:

**Principal constructs of interest:**

- GOCD: Discussion of overarching goals of care (i.e. values, goals, specific treatment preferences) that transcend beyond "just code status" and that reflect any exploration/discussion/verification during the *current hospitalization or clinic visit*.
- ACP/DPOA: Discussion of ACP document or DPOA decision with some sort of exploration, discussion, or verification during the *current hospitalization or clinic visit*.
- PROGNOSIS: Explicit documentation of patient's prognosis, or discussion thereof.

**Diversions:**

- SUBTHRESHOLD: References to goals of care, hospice, palliative care, etc. that don't contain meaningful content. Also used for mention of ACP/DPOA not explored/discussed/verified in the current hospitalization or clinic visit.
- LIVING IN THE PAST: Summaries of previous GOC discussions that took place prior to the current hospitalization or clinic visit, AND that that fail to reflect any re-exploration, discussion, or verification during the current hospitalization or clinic visit.
- JUST CODE STATUS: Discussion of the treatment preferences of intubation and cardiac resuscitation, without reference to patients' values, goals, or *any* other treatment decisions.

## GOCD: Documentation of goals-of-care discussions

**DEFINITION:** GOC discussions talk about "the overarching aims of medical care for a patient" for *when the patient becomes sicker or when they are dying.* This includes, but is not limited to, planning for future hospitalizations, worsening of disease, or end-of-life care. Ideally, a GOC discussion is always "informed by patients' underlying values and priorities," and may ultimately lead to or include "decisions about the use of, or limitation(s) on, specific medical interventions." However, many GOC discussions you will encounter focus closely on specific medical interventions. [Secunda et al, JGIM 2019]
Please use this code for documentation of discussions that pertain to the patient's overarching goals for care, including values/goals, understanding of illness, and specific treatment preferences that occurred during this hospitalization or clinic visit. Documentation of GOCD that were not necessarily carried out by the writer but occurred during this admission are also coded as GOCD.
Examples:

- "On 06/01, Dr. _______, as well as the patient's wife, daughter, and son in law had a family meeting. Some details included below, but agreement that in light of the patient's worsening clinical condition, it would be appropriate to transition to care focused more on patient comfort rather than longevity."
- "Please see my notes from 9/12 for full discussion. Briefly, she remains DNR/DNI and does not want to be intubated under any circumstances. She wishes for full care otherwise. If her medical condition worsens to the point that there are no other options, she would strongly consider transitioning to comfort-focused measures at that time."
- "GOC: See palliative care note from [3 days ago]. Family still wants all aggressive care for now but is considering comfort care."

Also use this code for documentation of code status decisions that reference any additional values, goals, or other treatment preferences. Examples:

- "Code status: Full (patient states she wants son to be able to tell her good bye)"
- "Code Status: DNR/DNI, do not escalate care"
- "Code Status: FULL CODE; ok with ICU, EGD, dialysis"
- "Code status: Full code, although patient would not want prolonged artificial support"

© 2023 Lee RY et al. *JAMA Network Open*.

Also use this code for documentation of discussions with patients/surrogates surrounding transitions to comfort care or hospice, as these typically imply consideration of a significant shift from "default" longevity-driven treatments. Since the words "comfort" and "hospice" are not always used consistently (is comfort a value? a goal? a treatment approach?), these passages would not necessarily rise to the level of the subcodes for GOCD-Values/Goals or GOCD-Treatments; however, they should still be coded as GOCD. Examples:

- "Patient's daughter (and LNOK) today discussed with attending Dr. ______ today the possibility of converting the patient to comfort care."
- "Patient interested in hospice."

Mere mention of "comfort care," "palliative care," or "hospice" without evidence of any actual discussion may be coded as SUBTHRESHOLD.

**★ Please tag all parts of the discussion that pertain to goals of care. (See above under "Coding practices" for more detail on how to code longer passages.)**

## → GOCD sub-codes

Within passages coded as GOCD, please use these sub-codes to tag any clear instances of the following three domains: understanding of illness, treatment preferences, and values/goals.

Sub-codes should be used only when the text ***jumps out at you with clarity*** in these domains.

### GOCD-Understanding: Documentation of **preexisting understanding/perspective re: illness**

***"What have doctors told you thus far?"*** This code aims to measure documentation of discussion about the patient or family's global understanding of and/or perspectives on the patient's illness. Assessing this understanding at the beginning of a GOC discussion helps align everyone.

Examples:

- "Mr. ___'s family understands that he is critically ill and at high risk of dying. They understand that his heart is no longer functioning well enough to support other organs."
- "Ms. ___ has been ill for a long time with worsening CHF and has long felt that this would be the disease that would lead to her death."
- "We discussed the patient's understanding of her illness and her desire to know the results of testing." (Documentation of discussion counts, even though the patient's understanding is not explicitly documented.)
- [I asked her what she heard about what is going on.] "She told me that she heard that she has problems with blood flow in her intestines and that it is causing her pain."

**Do not use** for "boilerplate legal" documentation that is devoid of all content.
Example that is NOT GOCD-understanding, if occurring in isolation:

- "Patient and family expressed understanding. All questions answered." (Understanding of what?)

### GOCD-Values/Goals: Discussions about **values and life goals**

Discussions with patients or surrogates about **what they think is most important in life**, or about **specific life results (goals) desired** by patients/surrogates. Patients might prioritize values such as life extension, independence, cognitive capacity, or comfort. They might express life goals of returning home, or living to see a child get married.

Examples:

- "Ms. ___ said that interacting with her family and her husband ___ are priorities. She does not want to suffer, but also prioritizes longevity enough to be willing to give up some comfort."
- "I talked with patient about his values regarding end-of-life care. He believes that life is worth living as long as his heart is beating."
- "Patient prioritizes his quality of life over quantity."

© 2023 Lee RY et al. *JAMA Network Open*.

- "Mr. ___ told us that he wants to be able to leave the hospital and go back to his farm."
- "Family hopes for patient to live long enough so that relative from Europe may visit next month."

### GOCD-Treatments: Discussions about **treatment preferences or limitations**

Discussions with patients/surrogates about **specific medical treatments**, such as hospitalization, intensive care, life-sustaining treatments, mechanical ventilation, CPR, dialysis, surgery, etc. May include preferences to receive, or to not receive, specific types of treatments.

Examples:
- "Patient desires no CPR, intubation or artificial nutrition."
- "At this stage, the patient would like aggressive treatment for illnesses, including a trial of mechanical ventilation. She is ambivalent about CPR vs DNR."
- "Mr. ___ has expressed his wish to continue chemotherapy, despite a high risk of potential complications."

### *Sometimes, treatment preferences and values/goals may overlap, or occur in the same sentence.*

Examples:
- "The patient remains very interested in any treatments that may prolong his life." [Can apply both subcodes to this quote, which reflects a preference for life-sustaining treatments and a value of life extension.]
- "Patient states that he would want to be kept alive using artificial measures as long as he is not disabled in the long term." [Can apply both subcodes to this quote, which reflects a preference for life-sustaining treatments and a value/goal of avoiding disability.]
- "Patient stated today that she would not want aggressive interventions if she were not able to interact with her family." [Can apply both subcodes to this quote, which reflects a preference for avoiding aggressive interventions contingent upon outcomes conflicting with the value of being able to interact with family.]
- "Patient stated that her goals were to be able to recognize and communicate with her family and that she would be okay with living in a nursing home needing help with ADLs for a short term, but not indefinitely." [Can apply code GOCD-Values/Goals to green portion, and GOCD-Treatments to yellow portion.]

## Examples that are NOT a GOC discussion:

- Citation of past ACP documents without confirmation or revision [Use code SUBTHRESHOLD.]
- Discussions that do not pertain to *overarching aims* for *when the patient is sicker or dying:*
    - "Goal of physical therapy is for patient to be able to walk up 4 stairs prior to discharge."
    - "Patient wishes to take fewer medicines and reduce pill burden. Will stop atorvastatin."
    - "Patient wants to go to Europe but is afraid of flying. Will prescribe propranolol."
- Shared decision-making about routine medical care:
    - "I discussed with pt whether to pursue prostate ca screening. After deliberation, he declined."
    - "We reviewed the risks and benefits of monitoring this lung nodule versus biopsying it. The patient and I agreed to defer biopsy for now and check a follow-up CT in 3 months."
- Routine documentation of consent prior to a surgery or procedure:
    - "The risks/benefits/alternatives to the procedure were reviewed; patient consented to surgery."

## Notes:

- Subcodes of GOCD should be reserved for instances when the defined concept "jumps out at you with clarity." Some GOC discussions may not contain any such passages.
- Subcodes of GOCD need only be applied within passages that are coded GOCD. Content representing a subcode that occurs outside of a GOC discussion need not be coded.

© 2023 Lee RY et al. *JAMA Network Open*.

## ACP/DPOA: New, revised, or verified ACP document or DPOA, or conversation re: same

Documentation that an advance care planning document was discussed with patient/surrogate, completed, verified, or revised during the current hospitalization or clinic visit). Advance care planning documents/forms include: advance directives (AD), healthcare directives, healthcare DPOA forms, living wills for healthcare (LW, LWHC), POLST, and others. Examples:

- “Completed POLST today.”
- “Completed POLST with patient. He would not want CPR, but would want limited interventions.”
- “POLST form given to patient, will bring back next visit.”
- “Patient and wife recently completed Living Will today and brought it in. We discussed patient preferences if his illness should progress.”
- “Patient was given a Five Wishes form which we discussed today and I encouraged her to bring this back at our next visit.”

If a decision-maker (DPOA) is being changed or reaffirmed, please apply code ACP/DPOA, regardless of whether the paperwork formalizing this change/reaffirmation has been completed. Example:

- “Patient still wants his children to be his decision-makers, as he thinks their relationship is improving.”
- “Patient would like to make sister her DPOA, contact SW to start paperwork.”

Examples that are **NOT** ACP/DPOA:

- “Patient's POLST, dated [2 years ago], specifies DNAR, Limited Interventions, Antibiotics for comfort.” [Use code LIVING IN THE PAST.]
- “Per POLST: DNR/DNI.” [Use code JUST CODE STATUS.]
- “POLST in chart.” [Use code **SUBTHRESHOLD]**

## PROGNOSIS: Explicit documentation of prognosis, including unknown/uncertain

Explicit documentation of patient’s prognosis, including if it is unknown/uncertain. Examples:

- “Discussed with patient today that the CT scan shows further metastasis and previous tumors have grown despite 3rd-line chemotherapy. Unfortunately, he is no longer responding to treatment and his life expectancy is limited.”
- “Patient asked about chances of recovery and we had a long talk about his prognosis and what the future holds.”
- “Discussed prognosis with patient.”
- “Critically ill, very poor prognosis.”
- “Prognosis unclear as there is little experience with this disease process.”
- “Estimated Prognosis: Oncologic work-up is ongoing.”

## JUST CODE STATUS: Documentation of code status only

Statement of code status, or documentation of GOC discussion in which the ONLY content is in reference to code status—i.e., treatment preferences for intubation and cardiac resuscitation. Examples:

- “Code status: DNR/DNI”
- “Patient currently full code pending further investigation of altered mental status and prognosis discussion with family.”
- “Code Status - Summary of Conversation: Patient said she would want chest compressions and to be intubated if needed while hospitalized.”
- “Patient expresses a firm desire to not be resuscitated were she to code, and her husband at bedside confirms these wishes.”
- “After discussion of resuscitation outcomes, the patient decided to make herself DNR/DNI.”

© 2023 Lee RY et al. *JAMA Network Open*.

Counterexamples with "too much information for JUST CODE STATUS": ANY additional information about the patient's values, goals, or other treatment preferences should be coded as GOCD. Examples of code status discussions that should be coded as GOCD include:

- "Code status: Full (patient states she wants son to be able to tell her good bye)"
- "Code Status: DNR/DNI, do not escalate care"
- "Code Status: FULL CODE; ok with ICU, EGD, dialysis"
- "Code status: Full code, although patient would not want prolonged artificial support"

## LIVING IN THE PAST: Summary of (or reference to) separately documented GOCD prior to this hospitalization or clinic visit

The goal of LIVING IN THE PAST is to capture instances wherein providers have reviewed a patient's goals from a prior hospitalization or clinic visit, and have summarized or referenced these past records, BUT have not actually re-explored, discussed, or verified this information during the current hospitalization or clinic visit.

This code should be used for content that refers exclusively to discussions and decisions made prior to the current hospitalization or clinic visit, such as past GOC discussions from prior admissions or from previous outpatient clinic visits.

Note that if the information is re-explored, discussed, or verified during the current hospitalization or clinic visit, please use other codes and code the passage as if the discussion took place in the present (GOCD, ACP/DPOA, PROGNOSIS, etc.). There is no need to apply the LIVING IN THE PAST code to such content, as the process of re-exploration/re-discussion/verification effectively brings the GOC content to the present.

Examples:

- "Patient was noted to be DNR/DNI during an admission [2 months ago] for COPD exacerbation, at which time she had met with palliative care and decided to continue to pursue all available treatments."
- "I had had a conversation with the patient during her last admission in January, at which time she had expressed the wish to prioritize longevity, but not if it involved a tracheostomy."

Counterexamples: Note that references to GOC discussions that occurred earlier in the same hospitalization should not be coded as LIVING IN THE PAST, because we consider the current hospitalization to be the "present." Such passages should be coded using GOCD, ACP/DPOA, PROGNOSIS, etc.

- "Based on GOC discussion with palliative care on 9/2, we are now transitioning to comfort care." [Use code SUBTHRESHOLD]
- "Appreciate palliative care consultation. Pt expressed goals of wanting to be at home with family in his remaining time and is not interested in pursuing life-sustaining treatments." [Use code GOCD and subcodes.]

**No need to verify separate documentation.** We can take the note writer's word for it.

## SUBTHRESHOLD: Text that appears code-able but is devoid of any actual content

Use SUBTHRESHOLD for excerpts that do not provide any meaningful information about GOC or related topics, or for citation of existing ACP/DPOA without re-exploration, discussion, or verification during the current hospitalization or clinic visit. Consult the coding flowchart for details.

Reference to GOC without any details about the content of the discussion, or decisions that were made:

- "GOC discussions/disposition planning ongoing."
- "Held family meeting this afternoon to discuss patient's quality of life and GOC."
- "Discussed longstanding values and GOC."
- "Discussed with patient whether palliative care consult might be helpful."

© 2023 Lee RY et al. *JAMA Network Open*.

Reference to GOC-related topics without documentation of any discussion:

- “Patient on comfort care.”
- “Palliative care consulted, appreciate recs.”

Reference to existing ACP/DPOA without exploration/discussion/verification during this hospital admission:

- “Completed POLST with outpatient PCP last year.”
- “DPOA is sister, ______.”

© 2023 Lee RY et al. *JAMA Network Open*.

## Section B: Codes that do not measure GOC discussions

**These codes are intended to help identify content that does not represent a GOC discussion, but that may contain similar verbiage to a GOC discussion.**

**★ If a passage bears any Section A code (e.g. GOCD, ACP/DPOA etc.), you do not need to apply any Section B codes.**

### CONGRESS: Talk of something codable with NO action

*All talk and no action!* When a note writer documents their aspirations, plans, musings, or recommendations to do something codable (hold GOC discussion, talk about / refer to hospice, etc.) **BUT** has not actually *done* anything, or even *talked about it* with the patient/surrogate.
Etymology of code: “What’s the opposite of progress?” “Congress.”
Examples:

- “Patient critically ill with poor neurological prognosis. Will need to visit with family re: GOC.”
- “GOC pending family meeting, likely in AM.”
- “Plan: Consider Palliative Care referral if pain is not managed.”
- “At next visit, will need to revisit with patient what his GOC are in light of severe illness.”
- “Recommend palliative care consultation given poor prognosis.” [Written by a consulting team, who are not actually placing the palliative care consult nor discussing it with the patient/family.]

### NEGATIVES: Statements that something codable did not occur

Clinician’s notation that a GOC discussion, ACP document, or other coded element did not occur.
Examples:

- “We did not discuss goals of care today.”
- “POLST forms was not available.”
- “Palliative Care referral was not introduced today.”
- “Patient does not want to discuss code status.”

© 2023 Lee RY et al. *JAMA Network Open*.

# Special circumstances and FYIs

## Code status notes

Whenever a provider enters a new code status order, they are prompted with this dialog box:

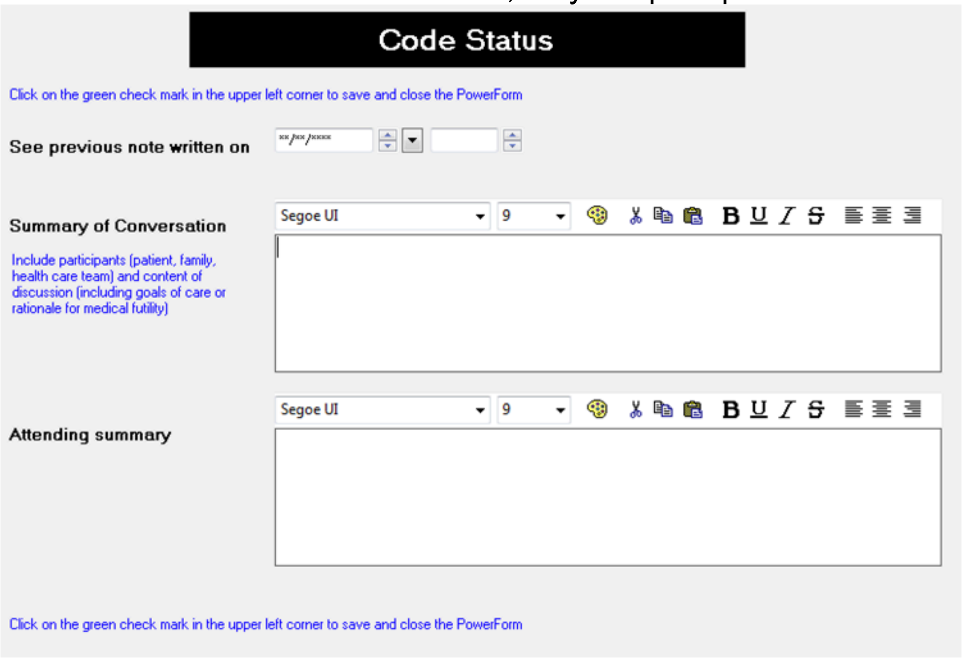


The responses in the dialog box auto-populate the following note in ORCA (text in italics is inserted by the system):

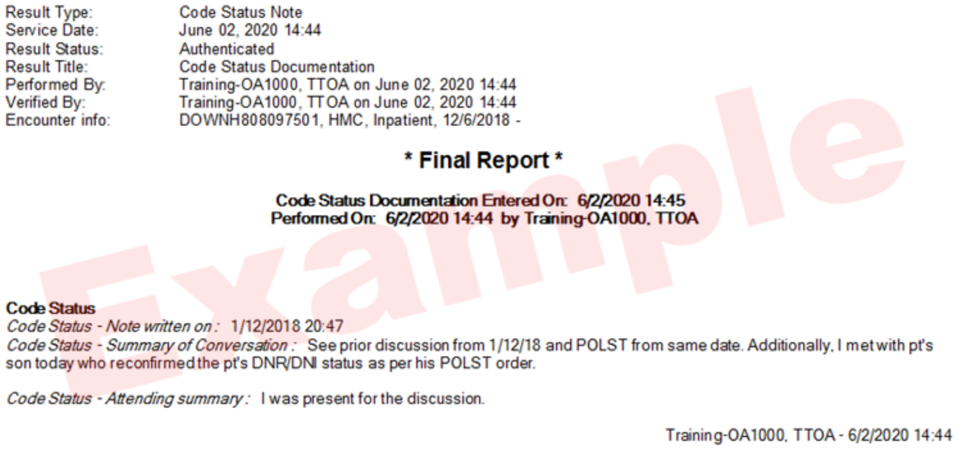

| | |
|---|---|
| Result Type: | Code Status Note |
| Service Date: | June 02, 2020 14:44 |
| Result Status: | Authenticated |
| Result Title: | Code Status Documentation |
| Performed By: | Training-OA1000, TTOA on June 02, 2020 14:44 |
| Verified By: | Training-OA1000, TTOA on June 02, 2020 14:44 |
| Encounter info: | DOWNH808097501, HMC, Inpatient, 12/6/2018 - |

*** Final Report ***

**Code Status Documentation Entered On: 6/2/2020 14:45**
**Performed On: 6/2/2020 14:44 by Training-OA1000, TTOA**

Example

**Code Status**
*Code Status - Note written on :* 1/12/2018 20:47
*Code Status - Summary of Conversation :* See prior discussion from 1/12/18 and POLST from same date. Additionally, I met with pt's son today who reconfirmed the pt's DNR/DNI status as per his POLST order.

*Code Status - Attending summary :* I was present for the discussion.

Training-OA1000, TTOA - 6/2/2020 14:44

In general, a code status note should be abstracted as if the free-text in the note were tacked onto the end of another note (e.g. an H&P).
Code the text at face value. It's okay if this results in duplicate coding of the same conversation (e.g. if you code GOCD for a brief description in the code status note that is in fact documented in more detailed fashion elsewhere).
Ignore the text "Code Status - Summary of Conversation" as this is auto-templated in by Cerner.

## Cerner fields

In the Cerner EHR system, an underscore ("_") is a field that a user can "jump" to by hitting F3. Underscores can therefore represent fields in note templates that were not filled out or checked off.

© 2023 Lee RY et al. *JAMA Network Open*.

## Cerner checklists

Some note templates contain checklists to simplify routine documentation. "[x]" means the writer checked off whatever is next to it; "[_]" means the writer left it unchecked. In some templates, the brackets are omitted. Please only code checked items.

## Attending attestations

Code attending attestations as if they were their own notes. In general, attending attestations should be coded as if they were independent notes. For example, when a GOC discussion is documented and the information is recapitulated in multiple sections of the same note (e.g. resident documentation, then attending attestation), please code each instance as if it were a unique GOCD.

## Imported ACP document lists

Apply <u>SUBTHRESHOLD</u> to imported lists of ACP Documents.
By default, discharge summaries and interim summaries contain a templated list of imported ACP documents (pictured below). Please apply code <u>SUBTHRESHOLD</u> to this documentation.

ADVANCE DIRECTIVE:

Patient has Advance Directive: No

List of Health Care Directives Scanned into UW Medicine Hospital Record:
Adv Dir Flowsheet 04/23/12
Adv Dir Flowsheet 09/11/14
POLST 02/21/15

## Palliative care data collection checklist

★ **Please ignore the palliative care data collection checklist** (pictured in part below):

ATTENDING TO COMPLETE (FOR DATA COLLECTION)

# Date consult requested: _

# Location at time of referral (choose one):
_Med/Surg
_Telemetry/Step Down
_Critical Care
_Emergency Department
_Acute Rehab
_Other: _

# Reasons given by referring provider for initial PC consult (chec
_GOC discussion/ACP
_Pain management
_Other symptom management
_Withdrawal of interventions
_Comfort care
_Support for patient/family
_Hospice referral/discussion
_No reason given
_Other: _
_Patient not seen because:

© 2023 Lee RY et al. *JAMA Network Open*.

**eAppendix 3.** Stata Source Code for Study Power Calculation and Simulation Procedures

```
/*  Power calculations and simulations for binary outcomes and exposures with non-differential misclassification
    -------------------------------------------------------------------------------------------------------------

    Robert Y. Lee, MD, MS (rlee06@uw.edu)

    Division of Pulmonary, Critical Care, and Sleep Medicine
    Cambia Palliative Care Center of Excellence at UW Medicine
    University of Washington School of Medicine

    References:
    - Mathematical approach to calculating power with outcome misclassification: Devine O. Eval Health Prof. 2003;26(3):315-39.
    - Stata parallelization library (to increase simulation speed): Vega Yon G, Quistorff B. https://github.com/gvegayon/parallel
      (type -ssc install parallel- to install this dependency; alternatively, un-comment non-parallelized code option in MCPowerSim_byLatentRD)

*/

version 17
set type double
// * parallelized code (comment out if running without -parallel- pkg):
   parallel setclusters 8

capture program drop MCPowerSim_singleSim
program define MCPowerSim_singleSim, rclass
    syntax, n1(integer) n2(integer) p1(real) rd(real) se(real) sp(real) alpha(real)

    // create 2x2 table of exposure by latent (unobserved, true) outcome
    clear
    set obs 4
    gen exposure=(_n>2)
    gen outcome_latent = (mod(_n,2)==0)

    // define cell values of 2x2 table based on provided assumptions (including assumption of Ha with specified RD between groups)
    gen _freq_latent = .
    replace _freq_latent = rbinomial(`n1', `p1')          in 2          // exposure==0, outcome==1
    replace _freq_latent = `n1' - _freq[2]                in 1          // exposure==0, outcome==0
    replace _freq_latent = rbinomial(`n2', `p1'+`rd')     in 4          // exposure==1, outcome==1
    replace _freq_latent = `n2' - _freq[4]                in 3          // exposure==1, outcome==0

    // simulate non-differential misclassification of latent outcome by its observed measurement
    // (i.e. convert 2x2 table to 2x2x2 table of correctly and incorrectly classified observations)
    gen _freq_misclassified = .
    replace _freq_misclassified = cond(`se'<1, rbinomial(_freq_latent, 1-`se'), 0) if outcome_latent==1
    replace _freq_misclassified = cond(`sp'<1, rbinomial(_freq_latent, 1-`sp'), 0) if outcome_latent==0
    expand 2, gen(_misclassified)
    label val _misclassified
    gen outcome_observed = cond(_misclassified, !outcome_latent, outcome_latent)
    gen _freq_observed = cond(_misclassified, _freq_misclassified, _freq_latent-_freq_misclassified)

    // expand dataset to 1 observation per patient
    expand _freq_observed

    // test for rejection of H0
    cs outcome_observed exposure
    return scalar H0_rejected = (r(p) <= `alpha')
```

© 2023 Lee RY et al. *JAMA Network Open*.

```
end
capture program drop MCPowerCalc_byLatentRD
program define MCPowerCalc_byLatentRD, rclass
    syntax, latent_rd(real) latent_p1(real) n1(integer) n2(integer) se(real) sp(real) alpha(real)

    local observed_p1 = `=`latent_p1'*`se'+(1-`latent_p1')*(1-`sp')'
    local observed_p2 = `=(`latent_p1'+`latent_rd')*`se'+(1-(`latent_p1'+`latent_rd'))*(1-`sp')'
    quietly power twoprop `observed_p1' `observed_p2', n1(`n1') n2(`n2') alpha(`alpha')

    di ""
    di "POWER CALCULATION WITH MISCLASSIFICATION:"
    di ""
    di "   Assumed, unobserved (latent, true) outcomes:"
    di "      p1 (latent): " %-06.4g `latent_p1'
    di "      p2 (latent): " %-06.4g `=`latent_p1'+`latent_rd''
    di "      RD (latent): " %-06.4g `latent_rd'
    di ""
    di "   Observed outcomes:"
    di "      p1 (observed): " %-06.4g `observed_p1'
    di "      p2 (observed): " %-06.4g `observed_p2'
    di "      RD (observed): " %-06.4g `=`observed_p2'-`observed_p1''
    di ""
    di "   Power, i.e. 1-beta given Ha and latent RD: " %-06.4g r(power)
    di ""

    return scalar observed_p1 = `observed_p1'
    return scalar observed_p2 = `observed_p2'
    return scalar observed_rd = `observed_p2' - `observed_p1'
    return scalar power = r(power)

end

capture program drop MCPowerSim_byLatentRD
program define MCPowerSim_byLatentRD, rclass
    syntax, latent_rd(real) latent_p1(real) n1(integer) n2(integer) se(real) sp(real) alpha(real) numRuns(integer)

    preserve
        di "Simulating power for n1=`n1' n2=`n2' latent_p1=`latent_p1' latent_rd=`latent_rd' se=`se' sp=`sp' alpha=`alpha' numRuns=`numRuns'"

// * non-parallelized code:
//      simulate H0_rejected=r(H0_rejected), reps(`numRuns') dots(`=round(`numRuns'/50)'): MCPowerSim_singleSim, n1(`n1') n2(`n2') ///
//          p1(`latent_p1') rd(`latent_rd') se(`se') sp(`sp') alpha(`alpha')
// * parallelized code (comment out if running without -parallel- pkg):
        parallel sim, expression(H0_rejected=r(H0_rejected)) reps(`numRuns'): MCPowerSim_singleSim, n1(`n1') n2(`n2') p1(`latent_p1') ///
            rd(`latent_rd') se(`se') sp(`sp') alpha(`alpha')

        ci prop H0_rejected
        return scalar powersim_95cilo = r(lb)
        return scalar powersim_95cihi = r(ub)
        return scalar power = r(proportion)
    restore

end
```

© 2023 Lee RY et al. *JAMA Network Open*.

```
// example execution code for calculation of, and Monte Carlo simulation of, study power at given latent risk difference, se, sp

local n1=1000
local n2=1000
local p1=0.25
local latent_rd=0.10
local alpha=0.05

local se=0.80
local sp=0.90

local numRuns=10000

MCPowerCalc_byLatentRD, latent_rd(`latent_rd') latent_p1(`p1') n1(`n1') n2(`n2') se(`se') sp(`sp') alpha(`alpha')
MCPowerSim_byLatentRD, latent_rd(`latent_rd') latent_p1(`p1') n1(`n1') n2(`n2') se(`se') sp(`sp') alpha(`alpha') numRuns(`numRuns')
```

© 2023 Lee RY et al. *JAMA Network Open*.

**eReferences**

101. Lee RY, Kross EK, Downey L, et al. Efficacy of a Communication-Priming Intervention on Documented Goals-of-Care Discussions in Hospitalized Patients With Serious Illness: A Randomized Clinical Trial. *JAMA Netw Open.* 2022;5(4):e225088.
102. Project to Improve Communication About Serious Illness—Hospital Study: Pragmatic Trial (PICSI-H, Trial 1). ClinicalTrials.gov identifier: NCT04281784. https://clinicaltrials.gov/ct2/show/NCT04281784. Accessed November 3, 2020.
103. Curtis JR, Lee RY, Brumback LC, et al. Improving communication about goals of care for hospitalized patients with serious illness: Study protocol for two complementary randomized trials. *Contemp Clin Trials.* 2022;120:106879.
104. Foley RN, Parfrey PS, Harnett JD, et al. Hypoalbuminemia, cardiac morbidity, and mortality in end-stage renal disease. *J Am Soc Nephrol.* 1996;7(5):728-736.
105. Goldwasser P, Mittman N, Antignani A, et al. Predictors of mortality in hemodialysis patients. *J Am Soc Nephrol.* 1993;3(9):1613-1622.
106. Owen WF, Jr., Lew NL, Liu Y, et al. The urea reduction ratio and serum albumin concentration as predictors of mortality in patients undergoing hemodialysis. *N Engl J Med.* 1993;329(14):1001-1006.
107. Soucie JM, McClellan WM. Early death in dialysis patients: risk factors and impact on incidence and mortality rates. *J Am Soc Nephrol.* 1996;7(10):2169-2175.
108. Zaslavsky AM, Beaulieu ND, Landon BE, et al. Dimensions of consumer-assessed quality of Medicare managed-care health plans. *Med Care.* 2000;38(2):162-174.
109. Fried LP, Tangen CM, Walston J, et al. Frailty in older adults: evidence for a phenotype. *J Gerontol A Biol Sci Med Sci.* 2001;56(3):M146-156.
110. Alsentzer E, Murphy JR, Boag W, et al. Publicly available clinical BERT embeddings. arXiv preprint arXiv:1904.03323; 2019.
111. Devlin J, Chang M-W, Lee K, et al. BERT: Pre-training of deep bidirectional transformers for language understanding. arXiv preprint arXiv:1810.04805; 2018.
112. Alsentzer E. BioClinicalBERT. 2019; https://huggingface.co/emilyalsentzer/Bio_ClinicalBERT. Accessed Mar 15, 2022.
113. Google Research. BERT: TensorFlow code and pre-trained models for BERT. 2018; https://github.com/google-research/bert. Accessed Mar 15, 2022.
114. Khalid S. BERT Explained: A Complete Guide with Theory and Tutorial. 2019; https://medium.com/@samia.khalid/bert-explained-a-complete-guide-with-theory-and-tutorial-3ac9ebc8fa7c. Accessed Dec 20, 2022.
115. Lee J, Yoon W, Kim S, et al. BioBERT: a pre-trained biomedical language representation model for biomedical text mining. *Bioinformatics.* 2020;36(4):1234-1240.
116. Lee J, Yoon W, Kim S, et al. BioBERT Pre-trained Weights. 2019; https://github.com/naver/biobert-pretrained. Accessed Mar 15, 2022.

© 2023 Lee RY et al. *JAMA Network Open*.
