# Supplementary material for: Assessment of Natural Language Processing of Electronic Health Records to Measure Goals-of-Care Discussions as a Clinical Trial Outcome
Source: JAMA Netw Open. 2023 Mar 2;6(3):e231204. doi: 10.1001/jamanetworkopen.2023.1204 (PMC9982698; doi:10.1001/jamanetworkopen.2023.1204)
Supplement: Supplement 2. — Data Sharing Statement [file jamanetwopen-e231204-s002.pdf]

Data Sharing Statement

Lee. Assessment of Natural Language Processing of Electronic Health Records to Measure Goals-of-Care Discussions as a Clinical Trial Outcome. *JAMA Netw Open*. Published March 02, 2023. doi:10.1001/jamanetworkopen.2023.1204

Data
Data available: Yes
Data types: Deidentified participant data, Other (please specify)
Additional Information: Due to the inherently-identifiable nature of textual electronic health records, we are unable to share textual EHR data, and are unable to share the fully-trained NLP model used in this study (which was trained on large quantities of identifiable protected health information).
How to access data: rlee06@uw.edu
When available: With publication

Supporting Documents
Document types: Statistical/analytic code
How to access documents: eAppendix 3 contains source code for misclassification-adjusted power calculations.
When available: With publication

Additional Information
Who can access the data: researchers whose proposed use of the data has been approved
Types of analyses: for any approved purpose
Mechanisms of data availability: with a signed data access agreement
